# Substrate Specific Inhibitor Designed against the Immunomodulator GMF-beta Reversed the Experimental Autoimmune Encephalomyelitis

**DOI:** 10.1038/s41598-020-60710-2

**Published:** 2020-03-02

**Authors:** Jane Jose Vattathara, Ohm Prakash, Sunitha Subhramanian, Madathiparambil Kumaran Satheeshkumar, Tessy Xavier, Meenakshi Anil, Gopal S. Pillai, Anandkumar Anandakuttan, Sureshkumar Radhakrishnan, T. B. Sivanarayanan, Unni AKK, Chethampadi Gopi Mohan, Krishnakumar N. Menon

**Affiliations:** 1 Centre for Nanosciences and Molecular Medicine, Amrita Institute of Medical Sciences and Research Centre, Amrita Vishwa Vidyapeetham, Ponekkara, Kochi-682 041 Kerala India; 2 Department of Neurology, Amrita Institute of Medical Sciences and Research Centre, Amrita Vishwa Vidyapeetham, Ponekkara, Kochi-682 041 Kerala India; 3 Department of Ophthalmology, Amrita Institute of Medical Sciences and Research Centre, Amrita Vishwa Vidyapeetham, Ponekkara, Kochi-682 041 Kerala India; 4 Central Animal Laboratory, Amrita Institute of Medical Sciences and Research Centre, Amrita Vishwa Vidyapeetham, Ponekkara, Kochi-682 041 Kerala India

**Keywords:** Computational models, Multiple sclerosis

## Abstract

The concept of substrate inhibition to prevent its phosphorylation has potential in drug discovery and is envisioned to treat the autoimmune disorder multiple sclerosis (MS). Glia maturation factor-β (GMF-β) Ser83 phosphorylation by protein kinase A (PKA) is pivotal in the activation of GMF-β-p38MAPK-NFκB biochemical pathway towards proinflammatory response induction in experimental autoimmune encephalomyelitis (EAE). Using structure-based drug design, we identified the small molecule inhibitor 1-H-indazole-4yl methanol (GMFBI.1) that specifically blocked Ser83 phosphorylation site on GMF-β substrate. Using *in vitro* and *in vivo* techniques, molecular mechanism of action of GMFBI.1’s direct interaction with GMF-β substrate and prevention of its Ser83 phosphorylation was established. GMFBI.1 down regulated p38MAPK phosphorylation and NFκB expression essential for proinflammatory response. Further, GMFBI.1 administration at peak of EAE reversed clinical symptoms, immunopathology, proinflammatory cytokine response and up regulated the anti-inflammatory cytokines. Present strategy of substrate inhibition against the key immunomodulatory target has immense therapeutic potential in MS.

## Introduction

Multiple sclerosis is a chronic autoimmune, demyelinating, neurodegenerative disorder of the central nervous system (CNS) affecting 2.5 million people globally^1,2^. Despite different disease-modifying therapies adopted to mitigate the inflammatory milieu of MS using different drugs^3–5^, patient’s progress from an acute phase to a stage with considerable neurological disabilities. Current therapies modulate disease differently as they target activated T cells, antigen presenting cells or prevent the egress of T cells into brain. Importantly, none of these drugs control molecules that modulate the proinflammatory response at a fundamental level following infiltration of activated T and B lymphocytes^6,7^. Activated lymphocytes undergo clonal expansion assisted by the cytokines produced mainly by astrocytes and microglia and glia maturation factor-β (GMF-β) plays an instrumental role in cytokine induction^8,9^. Thus, targeting GMF-β could signify a novel approach in controlling the immune response in the brain.

Over expression of GMF-β in response to immune challenge up regulates p38MAPK and NFκB expression in astrocytes leading to increased GM-CSF production by astrocytes and microglial generation of TNF-α, IL1-β, IL-6 and IFN-γ, augmenting proinflammatory response^10–12^. In the well-established EAE animal model of MS, GMF-β was over expressed in both brain and spinal cord^10^. On the other hand GMF-β null mice showed reduced mononuclear cell infiltration with a mild form of EAE compared to the regular EAE animals^10^. Administration of anti-GMF-β antibody to EAE animals led to reduced inflammation and clinical symptoms^13^. These studies imply the significance of GMF-β in controlling the specific proinflammatory response in astrocytes leading to the suppression of EAE. Thus, human GMF-β (hGMF-β) could serve as a potential therapeutic druggable target for inflammatory disorders like MS.

Phosphorylation of GMF-β on residues Thr27, Ser53, Ser72 and Ser83 by different protein kinases is critical in its activation and regulation^11,14^. PKA phosphorylates Thr27 and Ser83, while ribosomal S6 kinase, casein kinase and protein kinase C phosphorylates Thr27, Ser53, Ser72 residues respectively^14^. Among these, Ser83 phosphorylation of hGMF-β by PKA is crucial in inducing p38MAPK phosphorylation and activation of NF-κB leading to copious secretion of GM-CSF through GMF-β-p38MAPK-NFκB axis. Thus, controlling the protein kinases that phosphorylates hGMF-β residue at Ser83 is pivotal in regulating the proinflammatory response^14^. However, developing inhibitors against these generic kinases involved in the phosphorylation of hGMF-β is highly non-specific and could lead to adverse drug reactions. Thus, a novel molecular mechanistic strategy was conceptualized by which blocking the phosphorylation sites on hGMF-β substrate using small molecule inhibitors prevent the kinase mediated phosphorylation involved in GMF-β induced proinflammatory response.

Here, we report the identification of a naturally occurring small molecule [1-H indazole-4yl methanol] against hGMF-β substrate through structure-based drug design technique and named the compound as GMFBI.1. Further, validation of its inhibitory efficacy both *in vitro* and *in vivo* demonstrates its ability to suppress Ser83 phosphorylation of GMF-β and proinflammatory response leading to reversal of immunopathology in EAE animals. The present discovery shows the therapeutic potential of GMFBI.1 as a lead compound in modulating MS.

## Results

### Development of human GMF-β homology model and identification of active site residues involved in phosphorylation

Due to lack of the three dimensional (3D) structure of hGMF-β, homology modelling technique was employed to construct 3D structure of hGMF-β. We obtained murine GMF-β protein (PDB ID: 1V6F) structure solved using NMR technique as the best hit having BLAST score of 274, E-value of 1e-94 and sequence identity of 98%. The hGMF-β homology model development, its 3D structure quality analysis was given in (Supplementary, Fig. S1a–e**)**. Further, the 3D structural stability of hGMF-β for 40 ns molecular dynamics (MD) simulations is shown in Supplementary, Fig. S2.

Using SiteMap module, the active site residues of hGMF-β protein was predicted to be at SITE1 (Supplementary, Fig. S3**)**. It consists of key phosphorylating residues Thr27, Ser53, Ser72 and Ser83 of hGMF-β involved in the downstream signalling and proinflammatory response. These important observations further set the platform for structure-based drug design studies on this key neurological drug target.

### Human GMF-β model-based inhibitor design for blocking its phosphorylation sites to suppress the downstream signalling mechanisms

**Figure 1:**
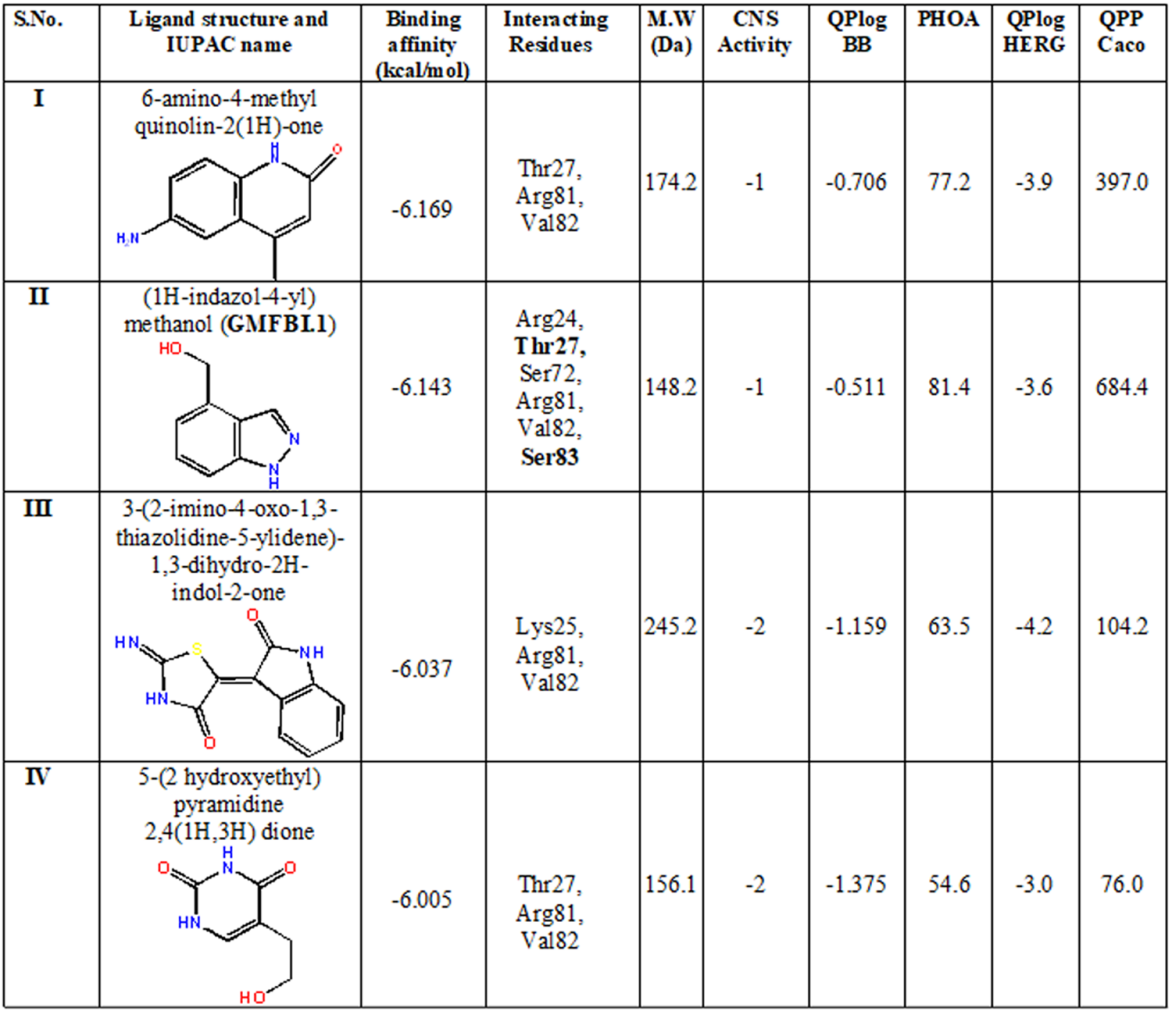
Ligand structure, binding affinity, interacting residues with human GMF-β and ADMET parameters of the VS hits. MW = Molecular weight of the molecule; CNS = predicted CNS activation on a –2 (inactive) to +2 (active) scale; QPlogBB = Predicted brain/blood barrier partition coefficient. Recommended values are from –3.0 to 1.2. None of the hit molecules go outside the recommended range; Percent Human-Oral Absorption (PHOA) = 0 to 100% scale. The prediction is based on a quantitative multiple linear regression model. This property usually correlates well with Human Oral Absorption, as both measures the same property. Recommended values: >80% is high, <25% is poor. None of the hit molecules showed poor value. QPlogHERG = Predicted IC50 value for blockage of HERG K+ channels (Cardiotoxicity). Value below–5 is of concern of cardiotoxic action. All hit molecules showed values more than -5, so is not of concern; QPPCaco = Predicted apparent Caco-2 cell permeability in nm/sec. Caco-2 cells are a model for the gut blood barrier. QikProp predictions are for non-active transport. Recommended values range from <25 -poor, >500 -great. None of the hit molecules showed poor value.

**Figure 2:**
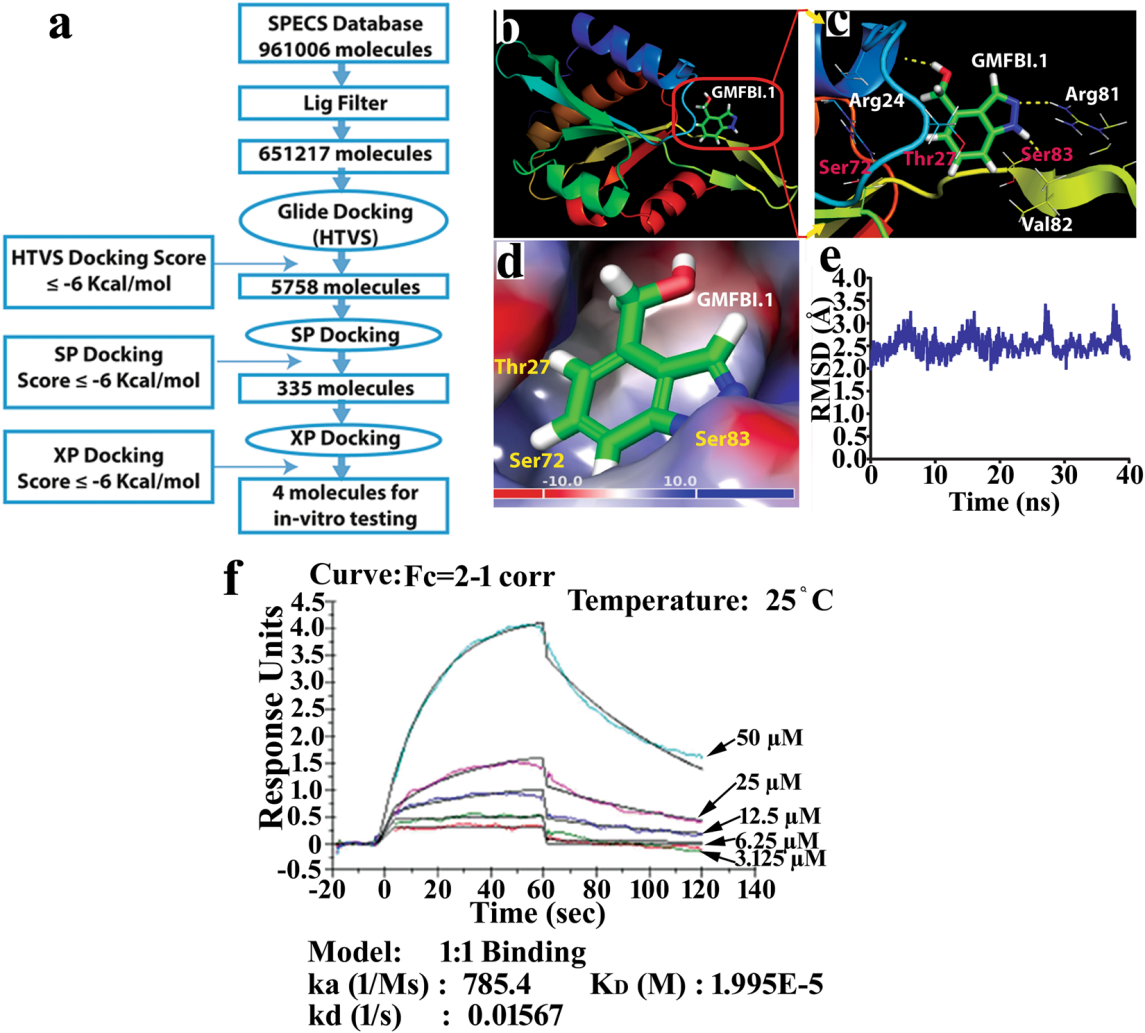
Sequential virtual screening (VS) strategy and the *in vitro* physical interaction between identified compound with GMF-β from SPECS database. (**a)** Structure based inhibitor design of GMF-β using high throughput virtual screening technique. **(b)** Human GMF-β in complex with the best docked pose of **GMFBI**.**1**. (**c**) Atomic level interactions view showing H-bond interactions between **GMFBI**.**1** and GMF-β via (i) Arg24, Arg81 and Val82 residues (Yellow dotted lines), (ii) phosphorylating residues Thr27, Ser72 and Ser83 (magenta color). **(d)** Molecular Electrostatic Potential **(**MEP) surface map of GMF-β in complex with **GMFBI**.**1** ligand. Maximum positive potential (+10 kcal/mol) and negative potential (−10 kcal/mol) depicted in blue and red color. **(e)** RMSD observed for 40 ns MD simulations of hGMF-β homology model depicts 3D structural stability. **(f)** Kinetics of compound GMFBI.1 binding with GMF-β protein is studied by SPR analysis using Biacore T200 system to obtain values for ka (association rate or on-rate), kd (dissociation rate or off-rate) and K_D_ (equilibrium constant of dissociation or affinity constant). hGMF-β protein binds to GMFBI.1 with micromolar affinity showing K_D_ of 19.9 µM.

Structure based drug design are well established in pre-clinical drug discovery programmes to evaluate the small molecule databases containing millions of compounds in order to identify new lead compounds towards druggable targets^15–20^. Sequential virtual screening (VS) strategies are applied to search the chemical space for potential compounds that binds to the active site of the hGMF-β protein by neglecting the false positive hits. Initially, LigFilter module was employed with respect to the physico-chemical properties of the successful CNS drugs. This VS step brought down SPECS database compounds from 961006 to 651217. The second step involves high-throughput VS of 651217 compounds using Glide module which filtered 5758 compounds on the basis of best docking pose and binding energies (BE ≥ −6 kcal/mol) of the compound with respect to GMF-β active sites. Further, simple precision (SP) based docking filtered of 335 compounds with BE ≥ −6 kcal/mol and finally extra precision (XP) docking method resulted in 4 best VS compounds and is shown in Fig. 1. In summary, the Glide docking results showed the highest scoring pose for each compound, and the ability to form H-bonding interaction with key residues at the active site gorge of hGMF-β (Figs. 1, 2).

Next, ADME/T properties of these compounds pertaining to pre-clinical drug discovery stages were analysed^21^. All the molecular properties predicted by the QikProp module of four VS hits (I-IV) are shown in Fig. 1. Compounds I and II had higher CNS activity relative to compounds III and IV. Mainly, compound II showed favourable gut to blood permeability (QPPCaco >500), high blood-brain barrier permeability (QPlogBB) and high oral absorption values (PHOA) relative to other three VS compounds. The compound II (10 mg/mL) has high aqueous solubility compared to IV (5 mg/mL in 10% DMSO), I and III are miscible only in 100% DMSO. Further, ADME/T profile of GMFBI.1 (compound II) was compared with few successful CNS drug like compounds reported in EAE (Supplementary, Table S1). GMFBI.1 toxicity profile was similar to drugs available in the market for the treatment of MS. Thus, the encouraging ADME/T properties and high aqueous solubility of GMFBI.1 favoured its selection for further *in vitro* and *in vivo* validation studies.

### GMFBI.1 binds directly to hGMF-β, inhibits PKA mediated phosphorylation of hGMF-β substrate *in vitro* and is non-toxic at lower concentrations on astrocyte cultures

**Figure 3:**
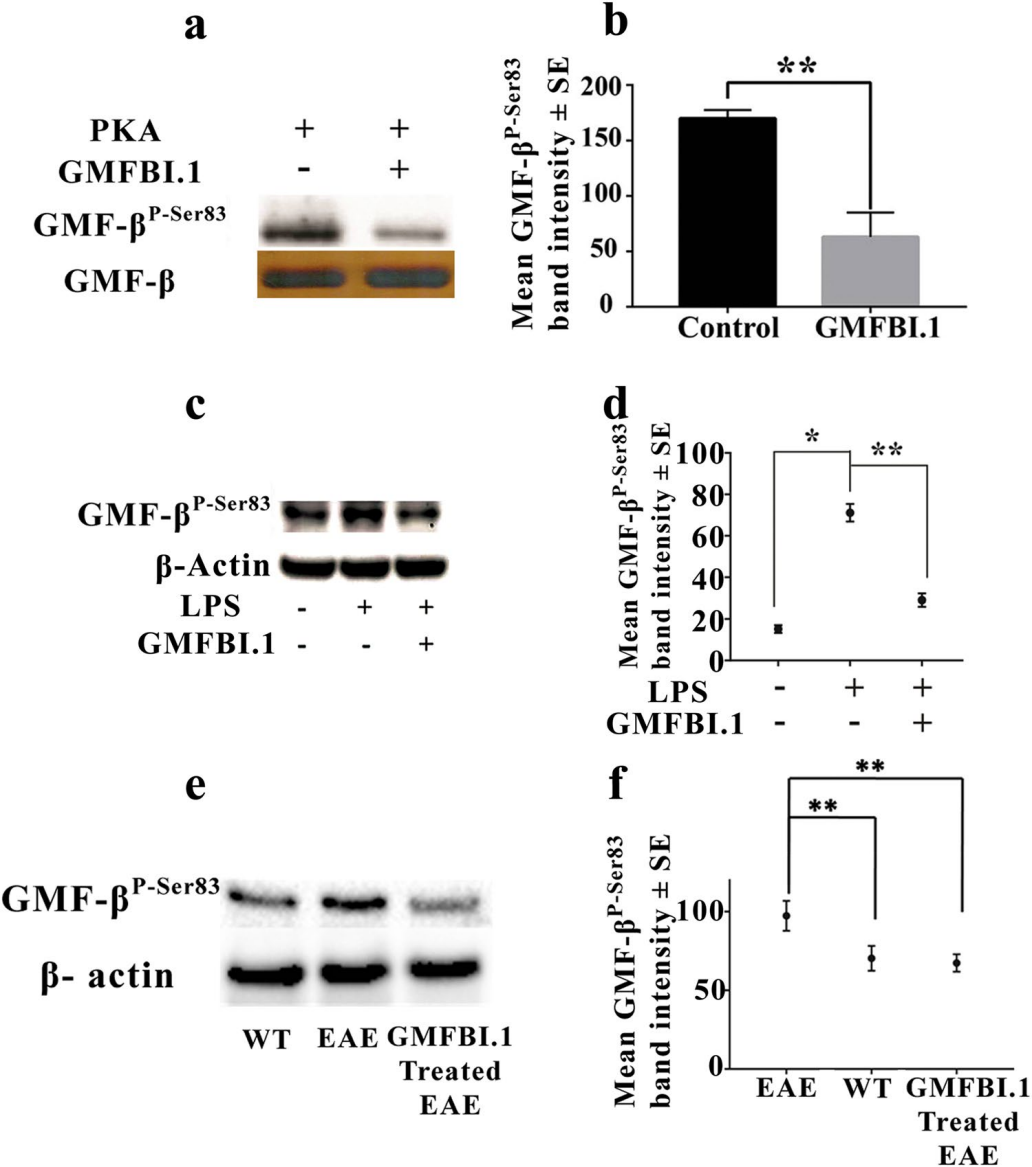
GMFBI.1 inhibits Ser83 phosphorylation of GMF-β. (**a)** 250 ng/mL of GMFBI.1. mediated inhibition of Ser83 phosphorylation of hGMF-β by PKA using purified hGMF-β and PKA **(b)** Note the significant reduction in Ser83 phosphorylation of GMF-β following GMFBI.1 compared to control (n = 3, **p < 0.01 vs vehicle alone). **(c)** LPS stimulated astrocytes were incubated with GMFBI.1 (500 ng/mL) for 30 min and analysed for Ser83 phosphorylation following immunoblotting using anti-phospho Ser83 antibody. **(d)** LPS treated astrocytes showed significantly elevated Ser83 phosphorylation compared to untreated control (*p = 0.0008). Note also the significant reduction in Ser83 phosphorylation on GMF-β following treatment with GMFBI.1 compared to untreated LPS control (n = 3, **p = 0.002). **(e)** *In vivo* inhibition of Ser83 phosphorylation of GMF-β in brain lysates following GMFBI.1 (12 mg/kg) treatment for 25 days. Note that Ser83 phosphorylation on GMF-βwas brought down to that of wild type brain lysates following administration of GMFBI.1 to EAE animals compared to untreated EAE animals. (**f**) Quantitative comparison of Ser83 phosphorylation on GMF-β in EAE vs WT (EAE vs WT; n = 3, **p < 0.01) and following treatment with GMFBI.1 in EAE (EAE vs GMFBI.1 treated; n = 3, **p < 0.01).

In order to further confirm the molecular mechanism of *in silico* binding of GMFBI.1 to hGMF-β, we performed surface plasmon resonance (SPR) based interaction studies. In a steady-state affinity 1:1 binding model, the affinity constant K_D_ value was 19.95 µM for GMFBI.1 towards hGMF-β and the residuals of kinetic data showed a Chi^2^ value of 0.0041 **(**Fig. 2f, Supplementary Fig. S4a,b). Subsequently, *in vitro* phosphorylation of hGMF-β using purified PKA and hGMF-β in presence and absence of GMFBI.1compound was done. Incubation of GMFBI.1 at 250 ng/mL (1.68 μM) significantly suppressed GMF-β phosphorylation on Ser83 compared to the vehicle alone control (*p* < *0*.*01vs control*) as judged by the specificity of the antibody to Ser83 phosphorylated 76–90 GMF-β sequence (Fig. 3a,b, Supplementary Fig. S5**)**. MTT toxicity assay of GMFBI.1 at different concentrations on astrocytes [0.1 to 3 mg/mL (670 μM to 0.2 mM)] showed astrocyte viability upto 1 mg/mL (6.70 mM) of GMFBI.1 (Supplementary, Fig. S6a**)**.

### GMFBI.1 mediated inhibition of p38MAP kinase activity and NFκB expression in astrocyte cultures

**Figure 4:**
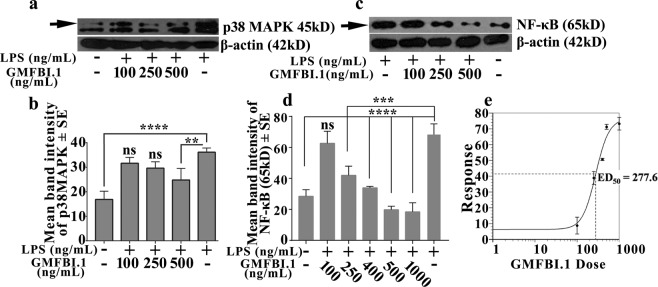
GMFBI.1 reduces p38MAPK phosphorylation and NFκB expression levels in GMF-β overexpressing LPS induced astrocytes *in vitro*. (**a)** Increasing concentrations of GMFBI.1 result in decreased p38MAPK phosphorylation. **(b)** At 500 ng/mL of GMFBI.1, statistically significant suppression of p38MAPK phosphorylation was noticed compared to untreated LPS stimulated cells (**p < 0.01). (**c**) GMFBI.1 suppressed the NFκB expression in a concentration dependent manner and was significant at 250 ng (****p < 0.0002), and in a range of 400–1000 ng (***p < 0.0001) compared toGMFBI.1 untreated LPS stimulated cells. (d) LPS treated vs untreated controls (***p < 0.0001). (**e**) ED_50_ of GMFBI.1 was calculated using the percentage mean band intensity of NFκB expression at various concentrations of GMFBI.1 to obtain the dose response. ED_50_ value of GMFBI.1 was found to be 277.6 ng/mL.

To verify the efficacy of GMFBI.1 in suppressing the proinflammatory cascade initiated by GMF-β, we over expressed GMF-β in astrocytes by stimulating with lipopolysaccharide (LPS) (Supplementary, Fig. S6b,c) and treated astrocytes with increasing GMFBI.1 concentrations (Fig. 4a,b). A gradual reduction in p38MAPK activity (Fig. 4a *(arrow)*, b) and suppression of NFκB (Fig. 4c *(arrow)*, d) expression were observed. At 500 ng/mL (3.36 μM) of GMFBI.1, statistically significant suppression of p38MAPK phosphorylation and NFκB expression were noticed (*p* = *0*.*0043 & 0*.*0001*) (Fig. 4d,e). Further, ED_50_ value was calculated to be 277.6 ng/mL by extending the observation with 400 and 1000 ng/mL of GMFBI.1 (Fig. 4d,e).

To further assess whether LPS mediated over expression of GMF-β and the effect of GMFBI.1 on Ser83 phosphorylation suppression can be reproduced in a GMF-β over expressing clone, we generated different stable clones of GMF-β by transfecting the human GMF-β construct into astrocytes and colonies were selected using G418 to make stable GMF-β expressing clones. As shown in Supplementary, Fig. S7a,b with increasing amounts of the GMF-β inhibitor GMFBI.1, a decrease in phosphorylation is seen and is found to be statistically significant (Fig. S7b).

### *In vivo* bio-distribution and toxicology evaluation of GMFBI.1

Having established the *in vitro* efficacy of GMFBI.1 in regulating GMF-β activity, we evaluated GMFBI.1 bio-distribution and toxicity *in vivo*. Intraperitoneal administration of 12 mg/kg of GMFBI.1 to C57BL/6 mice (n = 3) showed high clearance through the renal route and was non-toxic to the liver demonstrated by similar AST and ALT levels to that of control despite administering GMFBI.1 twice daily continuously for 25 days (Supplementary, Fig. S8a–d**)**. Importantly, GMFBI.1 could not be detected in any of the organs by 6^th^ hour including brain (Supplementary, Fig. S8a**)**. Absence of GMFBI.1 in brain prompted us to test the possibility of GMFBI.1 access to brain in EAE animals wherein blood brain barrier (BBB) is compromised^22^. Thus, we injected 12 mg/kg of GMFBI.1 i.p. in EAE animals with compromised BBB as in MS patients. Within 2 to 3hrs, maximum of 13 μg of GMFBI.1 could be seen in the brains of EAE animals and by 6^th^ hour, we failed to detect any GMFBI.1 in the EAE brains which is similar to the clearance of GMFBI.1 seen from all organs in normal mice as well (Supplementary, Fig. S8b).

### GMFBI.1 inhibitor reversed EAE and suppressed the proinflammatory response in severely paralyzed EAE animals

**Figure 5:**
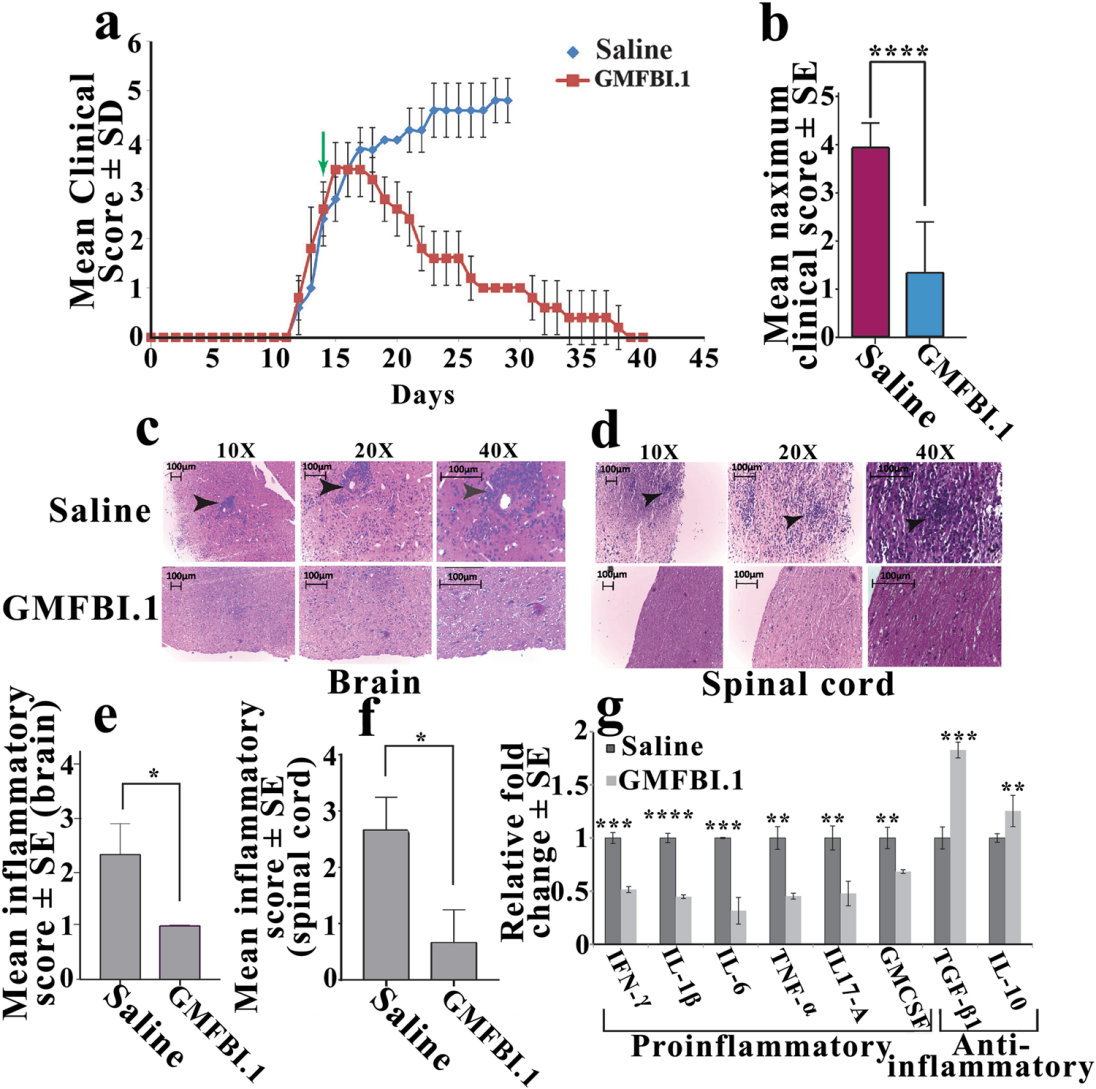
Reversal of EAE and associated immunopathology following treatment with GMFBI.1 in severely paralyzed EAE animals. (**a**) Clinical scores of GMFBI.1 (12 mg/kg i.p twice daily, arrow) and saline treated EAE (n = 5 per group) animals. **(b)** Note the significant reduction in the mean maximum clinical score following GMFBI.1 treatment compared to control (****p < 0.0001 GMFBI.1 treated vs untreated). (**c**) Assessment of mononuclear cells infiltration (arrows) in brain and (**d**) spinal cord by H&E staining in GMFBI.1 treated and untreated EAE animals. (**e**,**f**) mean histological score of (**c**,**d**) respectively (n = 3 per group). (**g**) Relative fold change ±SE of levels of cytokines following GMFBI.1 treatment normalized to the saline treated EAE controls in the sera of animals. Significant reduction in the proinflammatory cytokines levels and increase in anti-inflammatory cytokines in animals treated with GMFBI.1 could be seen compared to EAE controls (n = 3 per group) (*p < 0.05,**p < 0.01, ***p < 0.001, ****p < 0.001).

Based on the *in vitro* inhibitory effect as well as *in vivo* bio-distribution analysis of GMFBI.1, we administered 12 mg/kg of GMFBI.1 at day 14 twice daily to EAE animals at a clinical score 3. Within a week of treatment, the clinical score started to improve and by the second week, the animals were very much ambulatory. By day 40 post immunisation, animals demonstrated very low clinical score and regained its ambulatory capacity fully (Fig. 5a,b, Supplementary, Movies S1 vs S2). In conjunction with the clinical score reduction, significant down regulation of proinflammatory cytokines such as IFN-γ, IL-1β, IL-6, TNF-α, IL-17A and GM-CSF were seen in serum compared to vehicle treated controls and correlated with the reduced infiltration of mononuclear cells around the perivascular cuffs and reduced mast cell infiltration in the brain (Fig. 5c,d,g; Supplementary, Figs. S12, S14). This is manifested by decreased inflammatory score and significantly low levels of demyelination in brain and spinal cord of GMFBI.1 treated EAE animals compared to untreated EAE controls (Fig. 5e,f, Supplementary Fig. S13). Notably, the levels of anti-inflammatory cytokines TGF-β and IL-10 were significantly elevated in GMFBI.1 treated animals compared to vehicle treated controls (Fig. 5g). Importantly, the *in vitro* suppression of Ser83 phosphorylation by GMFBI.1 on GMF-β is recapitulated *in vivo* also, wherein, significant suppression of Ser83 phosphorylation on GMF-β was seen in the treated versus untreated EAE brains (*p* < 0.01) (Fig. 3e,f).

## Discussion

Present study unravels the molecular mechanism of action of a promising GMF-β substrate specific inhibitor in reversing the proinflammatory response propulsion in EAE through an integrated *in silico*, *in vitro* and *in vivo* approach. Blocking the GMF-β substrate Ser83 phosphorylation site rather than inhibiting the kinase involved in the phosphorylation led to down regulation of the key biochemical pathway involved in the propulsion of proinflammatory response and associated clinical symptoms, pathology and proinflammatory cytokine levels at the peak of EAE. Although similar concept of substrate blocking to prevent interaction between signal sequences of proteins with Sec. 61 involved in trafficking has been shown, the protein-protein interactions are of a different kind not involving a kinase^15^. Here, we developed a small molecule inhibitor GMFBI.1 using structure-based drug design strategy. GMFBI.1 blocked the key phosphorylating residue Ser83 of GMF-β, important in the downstream p38MAPK pathway involved in the propulsion of proinflammatory response (Figs. 2c,d, 3a–f).

Since human and mouse GMF-β phosphorylating residues are conserved, we solved the hGMF-β 3D structure by homology modelling due to the better choice for structure-based drug design studies by considering the potential translational aspects of the present study. Further, 3D structure quality of the hGMF-β model and MD simulation studies provided good structural accuracy making it suitable for structure-based blocker design **(**Supplementary, Fig. S2**)**. The best site map predicted residues (SITE-1) matched with the phosphorylating residues of GMF-β including the key Ser83 residue established earlier^11,14^ confirming the quality of our hGMF-β protein model **(**Supplementary, Fig. S3**)**. Subsequently, using Glide molecular docking methods, we identified four potent inhibitors against hGMF-β with BE ≥ −6 kcal/mol (Figs. 1, 2a*)*. The ADMET profiles of these compounds were assessed to provide insight into our *in vitro/in vivo* studies as shown for discovering best lead compounds in the pre-clinical drug discovery program^23,24^.

With the demonstration of GMFBI.1 binding to the active phosphorylation site of hGMF-β by *in silico* (Fig. 2c,d**)** as well as its direct physical interaction of molecules using SPR analysis (Figs. 2f, S4), we checked the efficacy of GMFBI.1 to block the PKA mediated Ser83 phosphorylation of hGMF-β *in vitro* as Ser83 is involved in p38MAPK activation cascade^14^. Indeed, significant reduction in Ser83 phosphorylation of hGMF-β by GMFBI.1 was noticed in cell free assay system, in cultures of astrocytes over expressing GMF-β and in EAE compared to control (Fig. 3a–f) indicating GMFBI.1’s high efficacy in blocking the predicted sites (Fig. 2b,c, Supplementary, Fig. S3**)**. Notably, GMFBI.1 establishes direct hydrogen bonding interaction with Ser83 residue (Fig. 2c). This could account for the high efficacy of GMFBI.1 in blocking the Ser83 phosphorylation seen both *in vitro* and *in vivo* (Fig. 3a–f).

**Figure 6:**
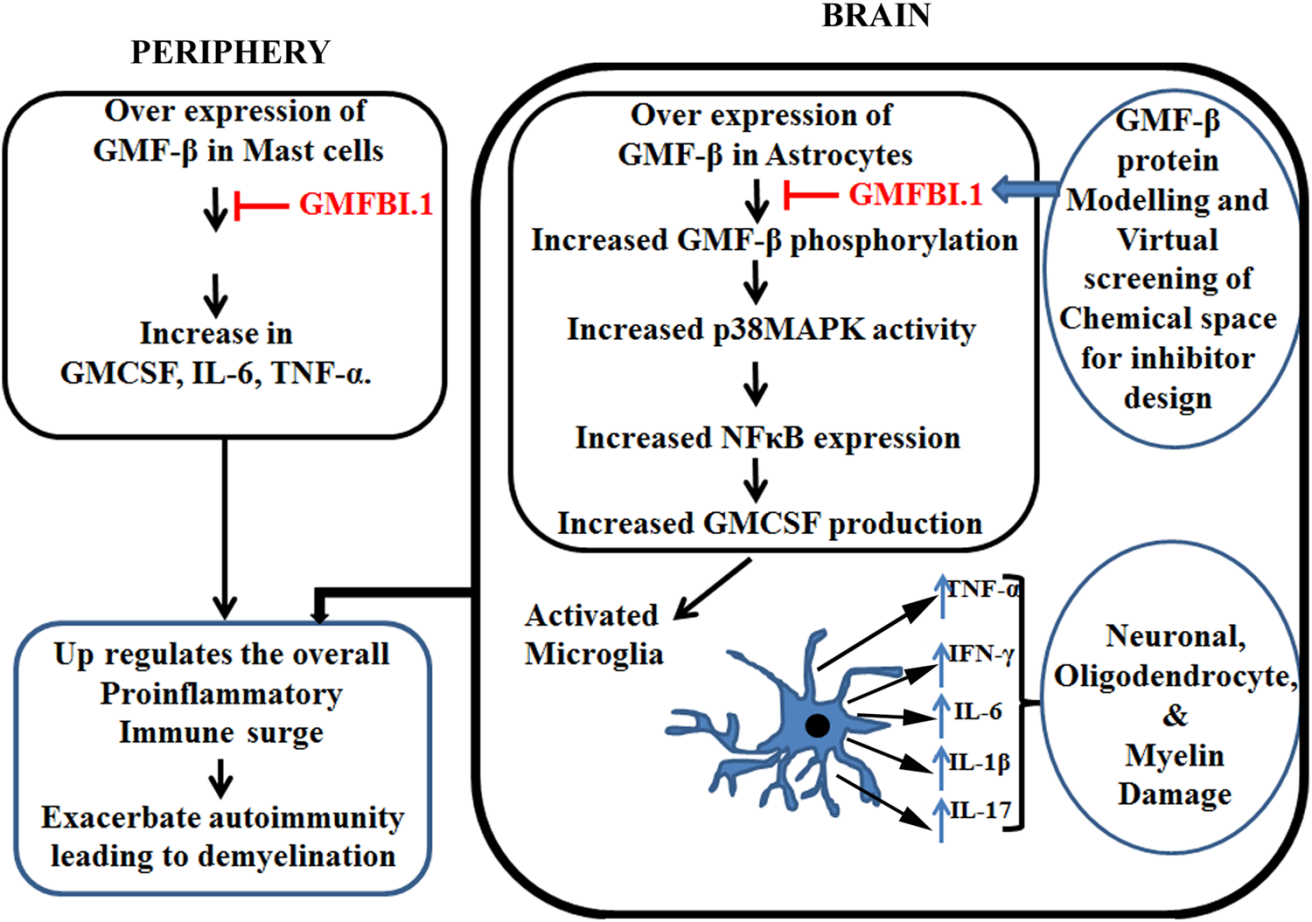
Molecular mechanism of action of GMFBI.1 compound mediated suppression of proinflammatory response by down regulating GMF-β mediated signalling pathway and cytokine production. Over expression of GMF-β results in up regulation of GMF-β phosphorylation by PKA leading to increased p38MAPK phosphorylation and activation of NFκB expression. This results in increased GM-CSF production by astrocytes resulting in microglial activation and secretion of different proinflammatory cytokines. Similarly in mast cells, the up regulation of GMF-β results in immune activation. The increased proinflammatory response both at the peripheral and CNS level could induce oligodendrocyte and neuronal damage. The lead compound GMFBI.1, prevents the overt phosphorylation on Ser83 residues of GMF-β resulting in down regulation of proinflammatory response.

Having shown the GMFBI.1 efficacy, we evaluated GMFBI.1 toxicity to astrocytes expressing GMF-β. Up to 1 mg/mL (6.5 mM) GMFBI.1 concentrations, the astrocytes remained viable **(**Supplementary, Fig. S6a**)** and that at 250 ng/mL (1.68 μM) concentrations, GMFBI.1 was effective in suppressing hGMF-β Ser83 phosphorylation (Fig. 3a,b**)** and remained well within 1 mg/mL of GMFBI.1 **(**Supplementary Fig. S6a**)**. The drop in Ser83 phosphorylation is accompanied by reduced p38MAPK phosphorylation and NFκB down regulation by GMFBI.1 in a concentration dependent manner (Fig. 4a–d). To further prove that GMFBI.1 effect is indeed through GMF-β, using hGMF-β expressing stable clones, we incubated different concentrations of GMFBI.1 with astrocytes clones expressing hGMF-β. A concentration dependent decrease in Ser83 phosphorylation of GMF-β indicates that effect of GMFBI.1 is through GMF-β (Supplementary Fig. S7). The suppression of GMF-β activity by blocking phosphorylation is crucial in the context of copious secretion of GM-CSF following activation of GMF-β-p38MAPK-NFκB axis (Fig. 6). It is interesting to note that another halogen substituted derivative of the base indazole scaffold reported by Moore *et al*. suppresses EAE by targeting estrogen receptor^25^. This derivative however showed only very weak interaction (−3.03 kcal/mol) to a different site other than GMFBI.1 binding site on hGMF-β (Supplementary, Fig. S9, Table S1). Note that GMFBI.1 has the ability to significantly suppress Ser83 phosphorylation on GMF-β (Fig. 3a–f). Thus, the substitutions on indazole scaffold determines its specificity like seen with GMFBI.1 towards Ser83 site (gorge) of hGMF-β (Fig. 2b,c, Supplementary Fig. S9).

Next, we checked the bioavailability of GMFBI.1 and it’s *in vivo* toxicity to liver by measuring AST and ALT levels in mice. Being hydrophilic, GMFBI.1 exhibited high clearance and within three hours following 12 mg/kg i.p injection, majority of the compound was found in urine **(**Fig. S8a**)**. Notably, we failed to detect GMFBI.1 in the wild type brain of C57Bl/6 mice suggesting the low BBB penetrability of GMFBI.1 **(**Supplementary Fig. S8a**)**. Interestingly, in EAE animals, approximately 13 µg of GMFBI.1 was found in the brain within three hours following its i.p injection due to the BBB breaching **(**Supplementary Fig. S8b**)**. Notably in our *in vitro* study, 500 ng/mL (3.36 μM) of GMFBI.1 at 100% bioavailability down regulated p38MAPK phosphorylation and NFκB expression significantly **(**Fig. 4a–d). Thus, the presence of 13 μg of GMFBI.1 in mice brain was approximately 24 fold higher than the required amount indicating its adequate availability in the brain to elicit an effective inhibitory effect on GMF-β. Notably, both AST and ALT levels remained similar to that of control following treatment with 12 mg/kg (twice daily i.p dose) of GMFBI.1 indicating its non-toxic nature. These *in vivo* observations prompted us to test its efficacy in modulating EAE.

Following induction of EAE in C57BL/6 mice using MOG^35-55^ peptide, at the peak of the disease with a clinical score of 3, the animals were given 12 mg/kg of GMFBI.1 twice daily i.p. for 25 days based on the biodistribution analysis. Within seven days of treatment, significant improvement in weight gain and clinical score was observed and by day 40, animals were very much ambulatory compared to the control saline injected animals **(**Fig. 5b; Supplementary, Figs. S10, S11, Movies S1 and S2**)**. Since, inhibition of Ser83 phosphorylation of GMF-β is crucial in the activation of proinflammatory cascade, we verified the phosphorylation levels in EAE animals brain treated with and without GMFBI.1. As shown in (Fig. 3e,f**)** EAE animals treated with GMFBI.1 of 12 mg/kg showed significant Ser83 phosphorylation reduction on GMF-β and that the levels of Ser83 phosphorylation remained similar to wild type. This consolidates our *in silico* and *in vitro* observations on the molecular mechanism of GMF-β phosphorylation blockage in an *in vivo* setting.

Considering the established role of mast cells in MS and EAE, it is welcoming to see GMF-β expression in these cells and has shown to influence the induction and severity of EAE^26–28^. Interestingly, IL33, a cytokine up regulated in MS was shown to increase GMF-β expression in mast cells^26^. Thus, GMFBI.1 could modulate the GMF-β activity in mast cells in addition to regulating GMF-β locally in the CNS astrocytes to alter the cytokine response in EAE animals (Fig. 6; Supplementary, Fig. S14). Notably, reduced mast cell infiltration in GMFBI.1 treated animals is seen along with decreased levels of mononuclear cell infiltration and reduced demyelination **(**Supplementary, Figs. S12–S14**)**. Indeed, improvements in clinical scores were matched with the reduced inflammation score accompanied by down regulation of proinflammatory cytokines IFN-γ, IL-17A and GM-CSF particularly in the serum **(**Fig. 5c–g**)**. This observation is in agreement with the studies on GMF-β null mice wherein, the levels of mononuclear cell infiltrates were drastically reduced in the brain parenchyma compared to wild type EAE animals^10^. It is well known that both in MS and EAE, IL17 and GM-CSF play pivotal roles in disease progression and severity during the active phase of the disease. Expression of these key inflammatory cytokines in the periphery plays a major role in the pathology and severity of MS and EAE^29,30^. IL-17A contributes immensely to CNS inflammation than the damage caused by Th1 class of cytokines^31^. Monoclonal antibody secukinumab against IL-17A suppressed EAE and found to attenuate IL-6 mediated proinflammation in primary human astrocyte^32^. Thus, significant IL-6 and IL-17A down regulation by GMFBI.1 could alter the pathogenesis in the course of the MS disease. Cytokine subsets such as IFN-γ, IL-6, TNF-α and IL1-β induces the activation of macrophage, increases ROS production and drive the homing of T cell into CNS leading to increased levels of IFN- γ, IL-6 and TNF-α transcripts in CSF and CNS tissue of EAE mice and MS patients^30,33,34^. Notably, GMFBI.1 significantly down regulated the levels of IL1-β, IFN- γ, TNF-α and IL-6 following treatment **(**Fig. 5g**)**. Although, the exact role of IL1-β in MS remains unclear, elevated levels of IL1-β transcripts are seen in CSF, brain and sera of MS patients^35^ and that IL-1β stimulate T-helper cells and astrocytes to promote EAE pathogenesis by compromising BBB integrity^36^. Thus, down regulation of IL-1β following GMFBI.1 treatment in EAE could contribute to the suppression of pathogenesis of EAE. Further, the pleotropic cytokine GM-CSF responsible for the recruitment of peripheral leukocytes to CNS during EAE^37^ was also down regulated significantly in the sera of GMFBI.1 treated animals (Fig. 5g). It is well known that GM-CSF gene deficient animals show resistance towards EAE induction^38^, and the administration of neutralising antibody GM-CSF (MOR103) to MS patients led to reduced lesion activity in the CNS^39^. This cytokine is also responsible for the differentiation of microglia to dendritic cells, a common antigen presenting cells in the CNS. Also, over expression of GMF-β led to increased production of GM-CSF in astrocytes that mediates the release of proinflammatory cytokines by microglia^9^. Thus, down regulation of GM-CSF mediated by GMFBI.1 have multiple effects on various immune cells which in turn down regulate the overall proinflammatory response in the CNS (Fig. 5e–g). It is to be noted that other inflammatory modulators such as nicotine also suppresses EAE^40^. Nicotine mediated NFκB suppression involves down regulation of p65/p50 subunits of NFκB and blocking its binding to DNA^41^. Treatment of RAW264.7 macrophages with nicotine did not show any alteration in p38MAPK phosphorylation^42^. Thus, suppression of proinflammatory activity upstream by GMFBI.1 works differently to nicotine though both inhibitors are converging in NFκB down regulation.

In conjunction with the suppression of proinflammatory cytokines, significant up regulation of anti-inflammatory cytokines TGF-β and IL-10 following GMFBI.1 treatment to EAE mice was seen **(**Fig. 5g**)**. These cytokines were down regulated in MS and are important in maintaining an inflammatory balance^43,44^. Of note, mice with B cells that do not produce IL-10 did not recover from EAE, indicating the importance of up regulation of IL-10 following treatment with GMFBI.1^45^. Thus, the action of GMFBI.1 in creating a shift in the cytokine profile levels from pro to anti-inflammatory scenario establishes its potential role as an anti-inflammatory agent against inflammatory damage in EAE.

In conclusion, using a novel molecular mechanistic approach, we targeted the critical phosphorylation site of GMF-β substrate involved in the proinflammatory response induction in EAE led to identification of a small molecule GMFBI.1 by structure based drug designing. GMFBI.1 binds directly to GMF-β and blocks its Ser83 phosphorylation pivotal in p38MAPK activation and NFκB expression associated with a key biochemical pathway involved in the proinflammatory response particularly mediated by astrocytes in the brain. The efficacy of GMFBI.1 in subduing the proinflammatory response and up regulation of anti-inflammatory response at the peak of the disease is further validated *in vivo* by reversal of EAE. Importantly, the integrated approach in developing an inhibitor brings in a novel molecular strategy by which a substrate specific inhibitor was developed without crystal structure and validated its efficacy in suppressing EAE. The identified GMFBI.1 could serve as a promising candidate therapeutic molecule for treating inflammatory disease affecting brain due to its high aqueous solubility and clearance. The oral delivery study of GMFBI.1 is currently underway to translate the finding. The present concept of developing an inhibitor against a substrate of an enzyme than the enzyme itself opens up avenues for developing new therapeutics.

## Materials and Methods

### Homology modelling of hGMF-β

The hGMF-β sequence (NP_004115.1) was retrieved from NCBI protein database. The sequence with best sequence identity, blast score and expectation value with that of hGMF-β was retrieved from PDB using BLAST-P program for identifying template for homology modelling. Homology model of hGMF-β was built using MODELLER 9v9 program^46^, a computer program that models the 3D structure of proteins and their assemblies by satisfaction of spatial restrains was used for building the homology model. The predicted 3D model was further evaluated using PROCHECK and ERRAT programs in SAVES server, to determine the stereo chemical quality of the structure^47^.

### Molecular dynamics simulation

Molecular dynamic (MD) simulation was carried out for the homology modelled structure of hGMF-β using AMBER12 software package in order to understand its dynamic structural and energetic stability^48^. First step include the topologic and coordinate files of the initial structure by LEaP module using ff99SB force field. The system was solvated in a rectangular box of TIP3P water molecules with a minimum solute-wall distance of 8 Å. Thus, the whole simulation system resulted in 10183 water molecules with dimensions of (18.774 × 18.774 × 18.774) Å^3^. The solvated system was neutralized by adding sodium ions and then subjected to energy minimization in two consecutive steps. The first 500 steps of minimization were performed by the steepest descent method. Conjugate gradient method was subjected for the next 1000 steps of minimization during which the harmonic restraints with a force constant of 500 kcal were applied to the hGMF-β structure. The following system was then subjected to move freely in the second minimization step in which 1000 steps by steepest descent and 2500 steps by conjugate gradient methods were adopted. The system was then subjected to initial equilibration for a time period of 20 ps by heating from 0 to 300 K. The temperature was regulated with the Langevin dynamics with a collision frequency of 1 ps^−1^ with a pressure relaxation time set to 2.0 ps. The periodic boundary condition was used in the NPT ensemble with Berendsen temperature coupling and P = 1 atm with isotropic molecular-based scaling. SHAKE algorithm was applied to restrain all the covalent bonds involving hydrogen atoms and the Particle-mesh Ewald was used to treat the long-range electrostatic interactions. The integral time step was set as 2 fs and non-bonded cut-off of 10 Å was used. Final step involve the production MD simulation for 40 ns time interval. The dynamic motion of trajectories and energy information were recorded at every 20 ps time interval. Finally, (root Mean Square Deviation (RMSD) of the dynamic atomic positions of hGMF-β protein were evaluated by analysing its MD trajectories. The potential energy of the hGMF-β was also calculated for 40 ns MD simulations.

### Protein active site prediction and virtual screening

The SITEMAP module of Schrödinger *v9*.*2* was used for the active site prediction of the developed hGMF-β model^49^. It uses novel search and analysis to generate information on the character of binding sites by identifying all the possible active sites on the protein. SPECS small compound database having 961006 compounds were used for sequential virtual screening (VS). Initially, compounds were prepared using the LigPrep module of Schrödinger v9.2. LigPrep adds hydrogen atoms, generates tautomers, ionization states, ring conformations, and stereoisomers, and produces the minimized 3D structures using OPLS2005 force field. Possible ionization states were generated at target pH 7.0 +/− 2.0 using EPIK module. The compounds were desalted and no tautomers were generated. For each ligand, single stereoisomers were generated by retaining the specified chiralities. Further, single low energy ring conformation for each compound was generated. The prepared ligands were subjected to VS criteria using the LigFilter module of Schrödinger v9.2. The compounds satisfying the criteria’s such as Molecular Weight ≤400, Number of hydrogen bond (H-bond) donors ≤3, Number of H-bond acceptors ≤7, Number of rotatable bonds ≤8 and LogP ≤5 were filtered as the first stage of VS. This VS criteria employed were based on the attributes of successful CNS drugs reported earlier^21^.

The molecular docking was performed using the GLIDE (Grid-based Ligand Docking with Energetics) module of Schrödinger v9.2. Initially, the receptor grid was generated from the Receptor Grid Generation panel of GLIDE module using the OPLS2005 force field. The molecular docking scoring grids were positioned by selecting the centroid of the selected residues from SITE1 predicted region as described above. All the ligands with length less than or equal to 14 was assigned for docking procedure. By default, GLIDE Score (GScore) multi-ligand scoring function was used to score the poses of the ligand to judge its binding affinity (or binding free energy- BE) towards the active site of hGMF-β. GScore has a steric-clash term and added buried polar terms to penalize the electrostatic mismatches between the protein-ligand complexes. GLIDE docking involves three choices of docking precision. Initially, a high-throughput virtual screening (HTVS) was performed followed by the Standard-precision (SP) docking and later Extra-precision (XP) docking procedure to filter best possible candidates^50^. HTVS is used when rapid screening of the large compound databases is required. After HTVS docking, the Dock Scores were analysed, and all the poses showing docking score (or free energy) ≤−5 kcal/mol were exported for SP docking. Ligand poses with high SP docking scores of ≤−6 kcal/mol was used as an input for XP docking. XP docking ensures better VS with negligible false positives filtering using accurate docking simulations. Finally, the QikProp module (*version 3*.*3*) was used for absorption, distribution, metabolism, excretion and toxicity (ADMET) prediction in the last stage of our VS procedure by selecting the best XP based high docking score compounds. This program is capable of predicting the physically significant descriptors and pharmaceutically relevant properties of a given set of drug like compounds. These pharmacokinetic properties include (i) predicted central nervous system activity on a –2 (inactive) to +2 (active) scale; (ii) Molecular weight; (iii) QPlogBB- Predicted brain/blood barrier partition coefficient (−3–1.2); (iv) Percent Human-Oral Absorption (PHOA)- Predicted human oral absorption on 0 to 100% scale (>80% high, <25% poor); (v) QPlogHERG- Predicted IC_50_ value for blockage of HERG K^+^ channels (Cardiotoxicity) (Below −5 not considered); and (vi) QPPCaco- Predicted apparent Caco-2 cell permeability in nm/sec gut blood barrier (<25 poor, >500 great).

### Surface plasmon resonance based interaction analysis between GMF-β and GMFBI.1 using Biacore 200

The binding affinity of GMFBI.1 to GMF-β was examined by SPR (Biacore 200 biosensor instrument; GE Healthcare Bio-Sciences). GMF-β (50 ug/ml in 10 mM sodium acetate pH5.5 buffer) was immobilized on sensor-chip CM5 (Series S, GE Healthcare Life Sciences) using amine coupling protocol with flow rate 10 µL/min for seven minutes GE Healthcare Life Sciences). Among the two flow cells used, one was immobilized with GMF-β protein at ~8000 RU and the other flow cell was used as reference. During immobilization, running buffer used is PBS (1x), pH 7.4 and immobilization buffer used is 10 mM Sodium acetate, pH 5.5. GMF-β protein is immobilized for final response of 3742.7 RU. Interaction of GMFBI.1 to GMF-β is studied in 1X PBS buffer containing 5% DMSO at 25 °C. Five different concentrations (3.125, 6.25, 12.5, 25 and 50 µM) of GMFBI.1 were prepared by 2 fold serial dilution of GMFBI.1 from 50 uM concentration in 1X PBS + 5% DMSO buffer (running buffer). Subsequently, these different concentrations (in increasing order) of GMFBI.1 were passed over the immobilized sensor chip in different cycles (as in multi-cycle kinetics assay format). Surface was regenerated using high flow rate of running buffer (PBS+5%DMSO). The GMFBI.1 was passed for 60 seconds over the sensor surface and dissociated for 60 seconds with buffer at a flow rate of 30 µl/min. A kinetic curve fitting was performed using T200 Evaluation Software version 3.1 (GE Healthcare Bio-Sciences). The sensorgrams of test flow cell (FC2) is subtracted from the sensorgrams of reference flow cell (FC1). The subtracted sensorgrams were evaluated using biacore T200 evaluation software version 3.1. The obtained sensorgrams are fitted to the 1:1 binding fit model. The kinetics data are evaluated based on statistical measurements provided by the biacore evaluation software like Chi^2^ and U-value.

### Reagents and antibodies

GMFBI.1 compound was characterized by NMR and HPLC with >95% purity **(**Supplementary Fig. S15**)** was purchased from Specs (Netherlands), custom made rabbit anti-phospho Ser83 specific antibodies to GMF-β conserved between mouse, rat and human sequence 76–90 (QHDDGRVS(p)YPLCFIF; Product YZ6909 is from YenZyme, USA), MOG 35–55 (MEVGWYRSPFSRVVHLYRNGK) peptide, hGMF-β construct and Endofectin^TM^ Max transfection reagent from GeneCopoeia (USA), Pertussis toxin from List biological (USA), *Mtb* H37RA from Difco (USA). GMF-β from Origene (USA). Haematoxylin, Eosin, Chemiluminescent solution Luminata Forte, PKA, Adenosine triphosphate (ATP), LPS [*E. coli* 0111:B4], complete Freund’s adjuvant and Toluidine blue stain were from Sigma Aldrich-Millipore (USA), DMEM-F12 from Gibco (USA), Fetal Bovine Serum (FBS), Penicillin/Streptomycin, Trypsin (0.01%) were from Invitrogen (USA), protease inhibitor cocktail was from cOmplete, Mini EDTA-free protease inhibitor tablets Roche (Germany), antibodies against GMF-β, NF-κB p65, p38MAPK and Luxol fast blue (LFB) stain kit were from Abcam (UK), β-actin from Santacruz (USA), AST and ALT kit from Aspen (India), mouse Cytokines Multi-Analyte ELISA Array Kit- Qiagen (Germany), Vybrant-MTT cell proliferation assay kit and kinase assay buffer were from Thermo Fisher Scientific (USA).

### Cell viability, p38MAPK/NFκB assay and *in vitro* cell free phosphorylation assay

CTX-TNA2 rat brain primary astrocyte cell line (CRL-2006, ATCC) were treated with GMFBI.1 at concentrations 0–3 mg/mL for cell viability assay. p38MAPK and NFκB assay was done on LPS (100 ng/mL) stimulated astrocytes with different GMFBI.1 concentrations (100, 250 and 500 ng/mL) for 48 hours and expression levels were detected by immunoblotting with anti-phospho p38MAPK and anti- NFκB antibody respectively. The band intensity was quantified^51,52^ and ED_50_ of GMFBI.1 was calculated from quantified NFκB expression levels against different GMFBI.1 concentrations including 400 and 1000 ng/mL for 48 h and values were plotted using AAT Bioquest® (USA) tool. *In vitro* phosphorylation of purified hGMF-β using PKA and GMFBI.1 was performed using 1 µg of GMF-β protein and 40U PKA in the presence and absence of 250 ng/mL of inhibitor GMFBI.1 suspended in the kinase assay buffer containing 10 mM ATP at RT overnight in a total of 20 µL assay volume. The kinase assay buffer was used as the diluent for 250 ng/mL of GMFBI.1. The control used include kinase assay buffer alone without the inhibitor. The phosphorylation reaction was stopped by adding SDS running buffer and subjected to SDS-PAGE and immunoblotted using custom made rabbit anti-phospho Ser83 antibody (1:1000) specific to the Ser83 residues of GMF-β and phospho GMF-β levels were quantified using *Image J* software from NIH, USA.

### EAE induction, clinical scoring, treatment, histological analysis and ELISA

Animal experiments were carried out using female C57BL/6 mice purchased from Tata Memorial Centre - Advanced Centre for Treatment, Research and Education in Cancer (ACTRAC), Mumbai with prior approval from the institutional animal ethical committee AIMS Kochi (Ref. No IAEC:2015/2/8). All animal experiments were carried out in accordance with institutional animal ethics guidelines and regulations. EAE was induced in female C57BL/6 mice (10–12 week old) using 200 µg of myelin oligodendrocyte glycoprotein (MOG) peptide [sequence 35–55; (MEVGWYRSPFSRVVHLYRNGK)] emulsified in complete Freund’s adjuvant supplemented with heat-inactivated Mycobacterium tuberculosis H37RA (4 mg/ml) subcutaneously. 200 ng pertussis toxin was given i.p. at day 0 and 2 post immunizations^53^. EAE scoring was done on a scale from 0 to 5 as follows: 1- tail weakness; 2-weakness in hind limb; 3- hind limb paralysis; 4- hind limb paralysis with fore limb weakness or paralysis; 5- moribund/deceased^38,53^. GMFBI.1 was administered in EAE animals at a stage of clinical score 3. 12 mg/kg of GMFBI.1 was dissolved in saline were administered twice daily, i.p for 25 days. Mice were monitored and scored daily for clinical symptoms and weight changes. For histology analysis, brain and spinal cord of EAE animals treated with GMFBI.1 and saline were harvested, fixed in 10% neutral buffered formalin. 5 μm thick paraffin wax embedded brain and spinal cord tissue sections were analysed after haematoxylin and eosin (H&E), LFB and toluidine blue staining^53–55^. Tissue morphology was analysed for mononuclear cell infiltration at the perivascular space and meninges, demyelination and for mast cell infiltration. Mean inflammatory score was plotted by single blind semi-quantitative analysis^56^. Further, several randomly distributed 20x fields of brain and spinal cord section areas of saline and GMFB1.1 treated mice (minimum n = 3 animals per group) were quantified for percentage area of mononuclear cell infiltration (H&E staining) and percentage area of myelination (LFB staining intensity) using *ImageJ* software.

### Serum cytokine analysis

Serum of GMFBI.1 and saline treated animals were analysed for the levels of Th1 & Th17 proinflammatory cytokines (IFN-γ,IL1-β,TNF-α, IL17-a,GM-CSF, IL-6) and Th2 anti-inflammatory cytokines (TGF- β and IL-10) after 25 days of treatment using enzyme linked immunosorbent assay according to the manufacturer’s instructions. O.D at 450 nm was read and relative fold changes with respect to saline treated were plotted.

### Statistical analysis

Data shown in this study are representations of at least three independent experiments and expressed as mean ± SEM. The band intensity from the blots is measured using *Image J* software and normalized to the actin bands for each blot to quantify the levels different bands analysed. Statistical differences between groups were evaluated by analysis of variance (ANOVA) with post hoc analysis (Dunnett’s test) and unpaired Students t-test using GraphPad Prism 7. Difference is considered statistically significant when *p* ≤ 0.05*.

### Data deposition

The 3D structure of modelled human GMF-β was deposited in Protein Model Database and assigned PMDB id: PM0081347.

## Supplementary information

Supplementary information

Supplementary information2

Supplementary information3
