# Supplementary material for: Substrate Specific Inhibitor Designed against the Immunomodulator GMF-beta Reversed the Experimental Autoimmune Encephalomyelitis
Source: Sci Rep. 2020 Mar 2;10:3790. doi: 10.1038/s41598-020-60710-2 (PMC7051966; doi:10.1038/s41598-020-60710-2)
Supplement: Supplementary file 1 — Supplementary information [file 41598_2020_60710_MOESM1_ESM.pdf]

SupplementaryMaterial

Substrate Specific Inhibitor designed against the immunomodulator GMF-beta Reversed the Experimental Autoimmune Encephalomyelitis.

Jane Jose Vattathara[†], Ohm Prakash[†], Sunitha Subhramanian[†], Madathiparambil Kumaran Satheeshkumar [†],Tessy Xavier[†], Meenakshi Anil[†],Gopal S Pillai[^], Anandkumar Anandakuttan[#], Sureshkumar Radhakrishnan[#], Sivanarayanan, T.B[+], Unni AKK[+], Chethampadi Gopi Mohan[†]*, Krishnakumar N. Menon[†]*

Centre for Nanosciences and Molecular Medicine[†],
Department of Neurology[#]
Department of Opthalmology[^]
Central Animal Laboratory[+]
Amrita Institute of Medical Sciences and Research Centre
Amrita VishwaVidyapeetham
Ponekkara, Kochi-682 041
Kerala, INDIA

Corresponding Authors*
Dr. Krishnakumar N Menon and
Dr. Chethampadi Gopi Mohan,
Center for Nanosciences and Molecular Medicine
Amrita VishwaVidyapeetham
Ponekkara, Kochi-682041
krishnakumarmenon@aims.amrita.edu
cgmohan@aims.amrita.edu

Supplementary Information Text

Methods

Cell Viability Assay

MTT (3-(4, 5-Dimethylthiazol-2-yl)-2,5-Diphenyltetrazolium Bromide) assay was performed to evaluate viability of CTX-TNA2 cells upon its exposure to GMFBI.1. Briefly, cells were trypsinized, seeded into 96 well plates and allowed to grow overnight. Different concentration of GMFBI.1 (0-3 mg/mL) were prepared in cell culture media and added into wells containing cells in triplicates. 10% triton X-100 containing media was used as control for cell lysis. After 48 hours of incubation with GMFBI.1, MTT reagent was added according to manufacturer's protocol. Optical density (O.D) was measured at 570nm using Beckmann Coulter Elisa plate reader (BioTek Power Wave XS).

In vivo phosphorylation assay and detection of Ser83 phosphorylation on GMF-β.

In vivo GMF- β phosphorylation levels were analysed in EAE, GMFBI.1 treated and wild type mice brains. Brains were excised and lysed in 0.32 M sucrose solution with sodium pyrophosphate (50 mM), β-glycerophosphate (50 mM) as phosphatase inhibitors and protease inhibitor cocktail using Macs (Miltenyi biotech) tissue homogeniser. 20μg of protein was loaded for SDS-PAGE and western blotting. The difference in the levels of GMF-β phosphorylation was analysed using custom made anti-phospho-Ser83-GMF (pSer83) polyclonal antibody. This is followed by chemiluminiscent reaction as per manufacturer's protocol. The phosphorylation signal was captured using Bio-Rad chemi doc imaging system and quantified using *Image J* software.

Transfection of CTX-TNA2 cells for hGMF-β overexpression

CTX TNA2 cells were transfected with vector pReceiver-M01 containing hGMFβ construct using Endofectin^{TM} Max Transfection Reagent according to manufacturer's protocol in Opti-MEM reduced serum medium. After 48 hours of transfection, the cells were cultured in medium containing G418 (7mg/ml) (Roche, USA) for 20 days for selection. The G418 resistant clones were then selected, and expression of hGMF-β was examined by Western blotting with anti-GMF-β antibody. Three clones expressing hGMF-β were selected further for GMFBI.1 incubations to analyse the potency of GMFBI.1 to inhibit Ser83 phosphorylation on hGMF-β. GMFBI.1 250 and 500 ng were incubated with the three different clones for 30 mins. The phosphorylation reaction was stopped by adding

SDS running buffer and subjected to SDS-PAGE and immunoblotted using custom made rabbit anti-phospho Ser83 antibody (1:1000) specific to the Ser83 residues of hGMF-β.

Dot-Blot assay to validate the specificity of phospho-Ser83 GMF specific (pS83) polyclonal antibody

Dot blot was performed with specific anti-phospho-Ser83 GMF-β polyclonal antibody against Ser83 phosphorylated 76-90 peptide fragment of GMF-β and the non-phosphorylated GMF-β 76-90 peptide fragment. Both these peptides were dot blotted at different concentrations on a nitrocellulose membrane according to manufactures protocol in Bio-Dot-SF microfiltration apparatus (Bio-Rad). Subsequently, the membrane was blocked with blocking buffer and probed with anti-phospho-Ser83-GMF-β specific polyclonal antibody. The phosphorylation signal was captured using Bio-Rad chemi doc imaging system

Bio-distribution Analysis

For bio-distribution analysis, female C57BL/6 mice of 8-10 weeks (n=3) were injected i.p either with GMFBI.1 (12 mg/kg) or saline. Mice were euthanized by CO_2 asphyxiation at different time points (0.5, 1, 2, 3 and 6 hours). Blood was collected via cardiac puncture and plasma was separated and stored. Further, brain, liver, lung, kidney and spleen also were harvested and stored for analysis. Urine was collected at (1, 2 and 3 hours) time frame after GMFBI.1 administration and analysed. For HPLC analysis, the various organs were first weighed and homogenised using a tissue homogeniser (Macs, Miltenyi biotech) in PBS. GMFBI.1 was then extracted from tissue homogenate by precipitating out tissue proteins using acetonitrile/water (3:1) mixture at -20°C overnight. Samples were centrifuged at 5000 rpm for 10 min and the supernatant was used for analysis of GMFBI.1 in LC-20AD-prominence HPLC (SHIMADZU) equipment. C18 column (Qualisil BDS C18 chromatography column, 5 µm, 4.6 × 250 nm) was used for GMFBI.1 analysis. Standards were prepared by measuring AUC of known concentration of GMFBI.1 in plasma precipitated using acetonitrile and water (10 mM ammonium bicarbonate) mixture at 214 nm. Retention time for GMFBI.1 was found to be 3.0± 0.8s at a flow rate of 1 mL/min. The amount of GMFBI.1 in each organ was then quantified from the standard plot.

Liver Function Test

For liver function test, female C57BL/6 mice of 6-10 weeks (n=3) were injected i.p either with GMFBI.1 (12 mg/Kg) or saline twice daily. Mice were euthanized by CO_2 asphyxiation at different time points (3, 10 and 25 days) and blood was collected via cardiac puncture immediately. Blood

serum was separated and levels of aspartate aminotranferase (AST) and alanine aminotranferase (ALT) were analysed by modified International Federation of Clinical Chemistry methods in Mindray BA-88Auto-chemistry analyser.

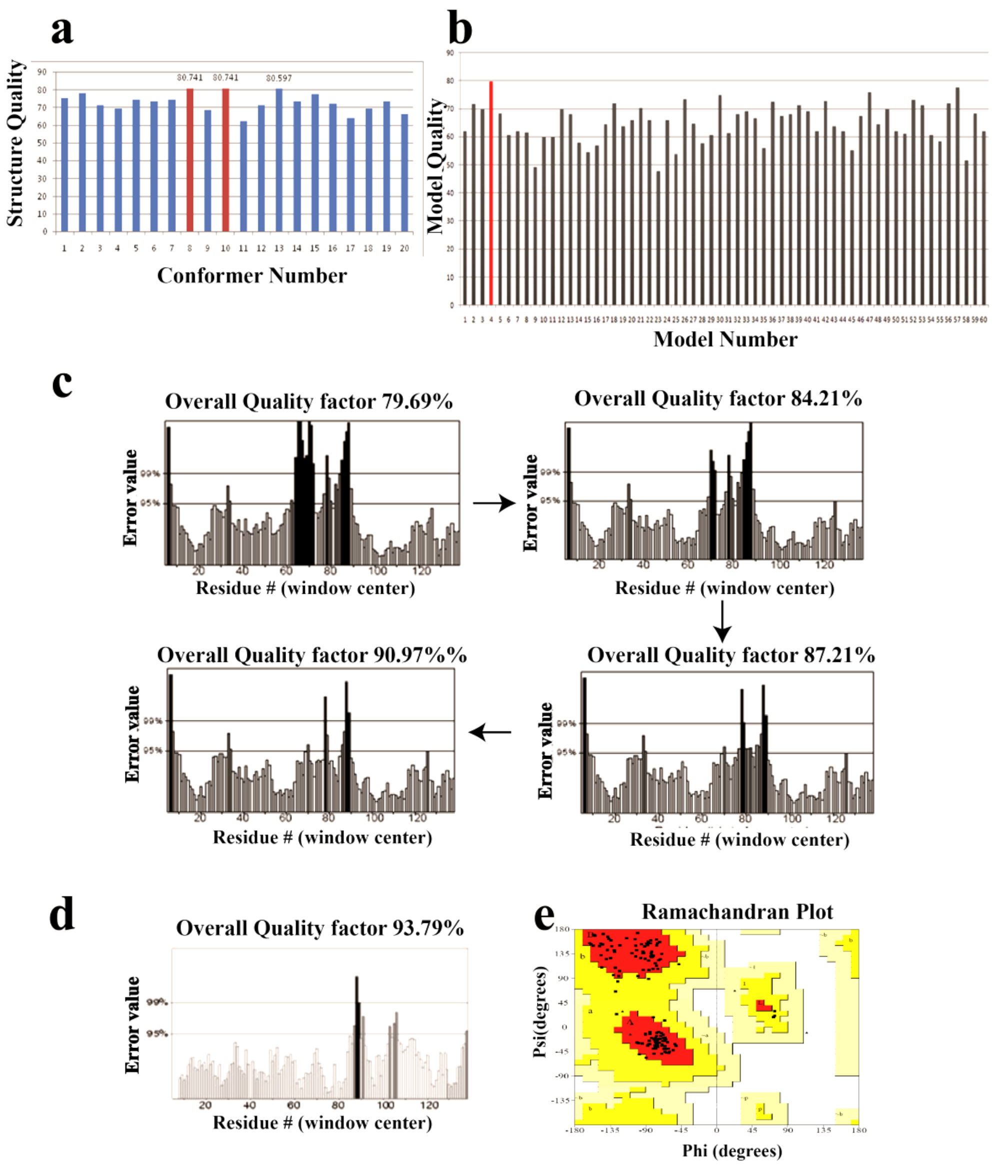

Figure S1. (a) NMR structure of murine GMF-β protein (PDB ID: 1V6F) consists of an ensemble of 20 different conformers. We analysed the structure quality of each of these 20 different NMR based conformers by ERRAT plot and 8th conformer predicted maximum structure quality of 80.74%. **(b)** This was chosen as the template structure for constructing the homology model of human GMF-β. Using MODELLER 9v9 software, 60 models of human GMF-β were built and 4th model was selected as the best model on the basis of PROCHECK and ERRAT program. The best model had an ERRAT structure quality factor of 90.97% **(c)** and the DOPE score was evaluated to be-15578.97. This was much better than the best DOPE score (-15300.69) amongst the initial 60 model structures generated as explained above. **(d)** Human GMF-β 3D structure quality was further improved to 93.8% by energy minimizing using OPLS2005 force field implemented in PrepWiz module of Schrödinger software.**(e)** Further, its Ramachandran map showing the stereochemical quality and overall 3D structural geometry was generated.

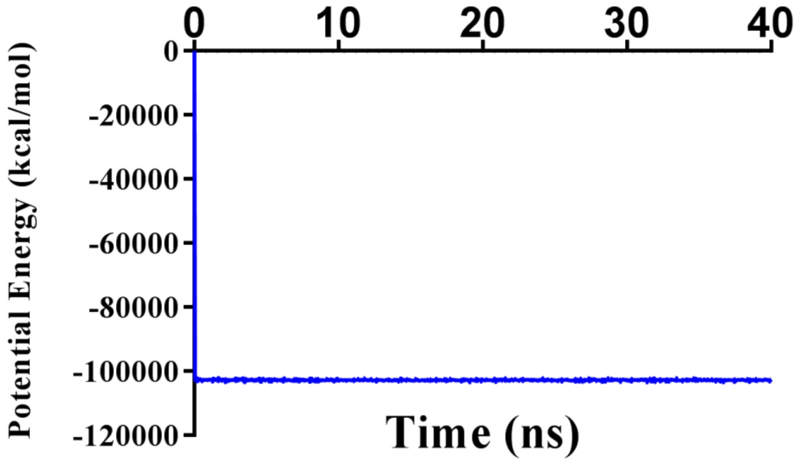

Figure S2. Potential energy of the hGMF-β for 40 ns MD simulations.

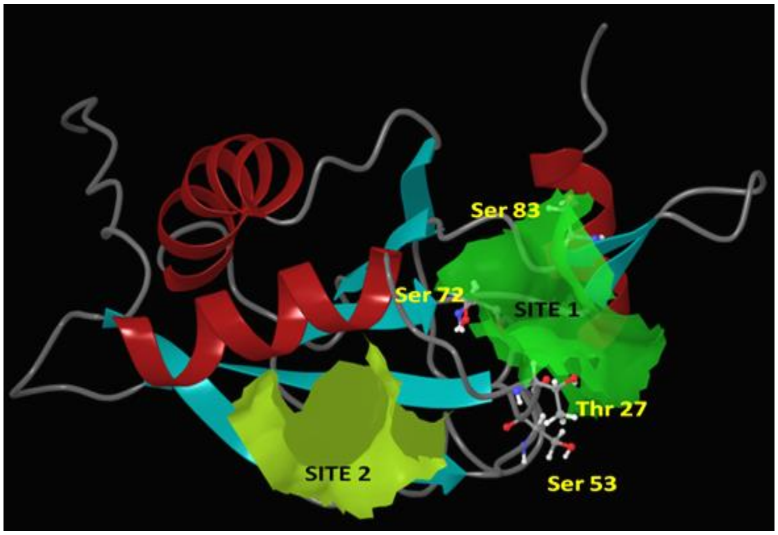

Figure S3. SiteMap predicted potential active sites (SITE 1, SITE 2) of hGMF-β obtained by SiteMap module. Best predicted site SITE 1, includes the residues- Thr27, Ser53, Ser72 and Ser83, and which is in accordance with its experimentally reported phosphorylating site residues.

a

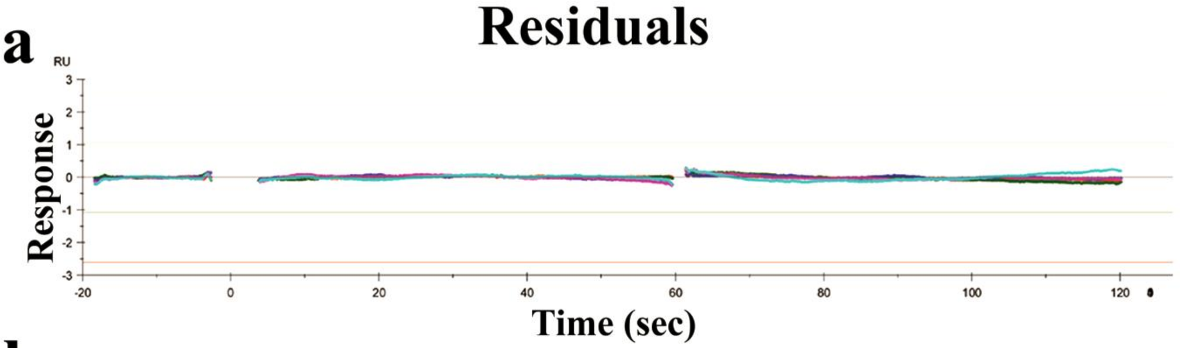

b

Sample	ka (1/Ms)	kd (1/s)	KD (M)	Rmax (RU)	Chi² (RU²)	Ligand	Model	Curve
GMFBI	7.85e+02	1.57e-02	1.99e-05	Local Fit	0.00411	GMFB protein_50ug/ml	1:1 Binding	Fc=2-1 corr

Figure S4. Kinetic screen of GMFBI.1 interaction to GMF-β using1:1 binding model. (a) Residuals showing the extent of data matching with the mathematical prediction. **(b)** The K_D value showing the dissociation constant equilibrium between hGMF-β and GMFBI.1 to be 19.9µM with a Chi^2 value of 0.0041 indicating the goodness of fitting.

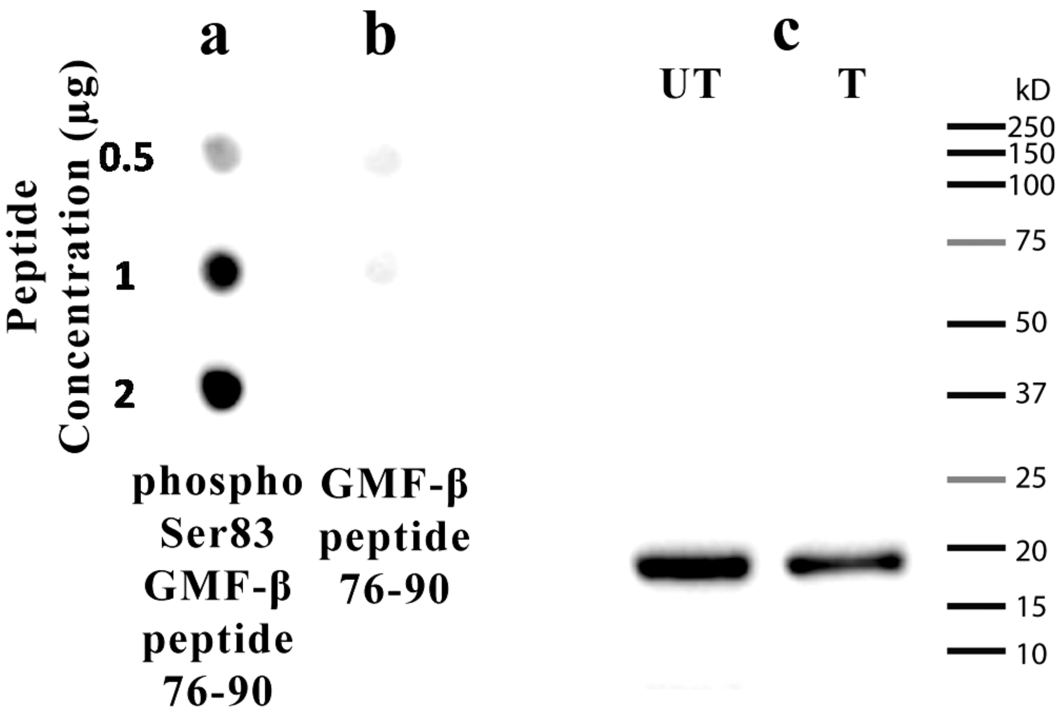

Figure S5. Dot-Blot assay to validate the specificity of phospho-Ser83 GMF-β specific polyclonal antibody against Ser83 phosphorylated 76-90 peptide fragment. GMF-β Ser83 phosphorylated 76-90 peptide fragment and the non-phosphorylated GMF-β 76-90 fragment were spotted on nitrocellulose membrane at different amounts as shown and probed to analyze the specificity of the antibody towards Ser83 phosphorylated GMF-β. **(a)** Note the intense specific reactivity of anti-phospho Ser83 polyclonal antibody to phosphorylated Ser83 76-90 GMF-β peptide compared to **(b)** the unphosphorylated 76-90 GMF-β peptide. (**c)** Brain lysate from EAE animals treated (T) and untreated (UT) with GMFBI.1were immunoblotted with anti-phospho Ser83 antibody. Note the specific staining of Ser83 phosphorylated GMF-β in the whole blot indicating the specificity of the antibody towards the GMF-β.

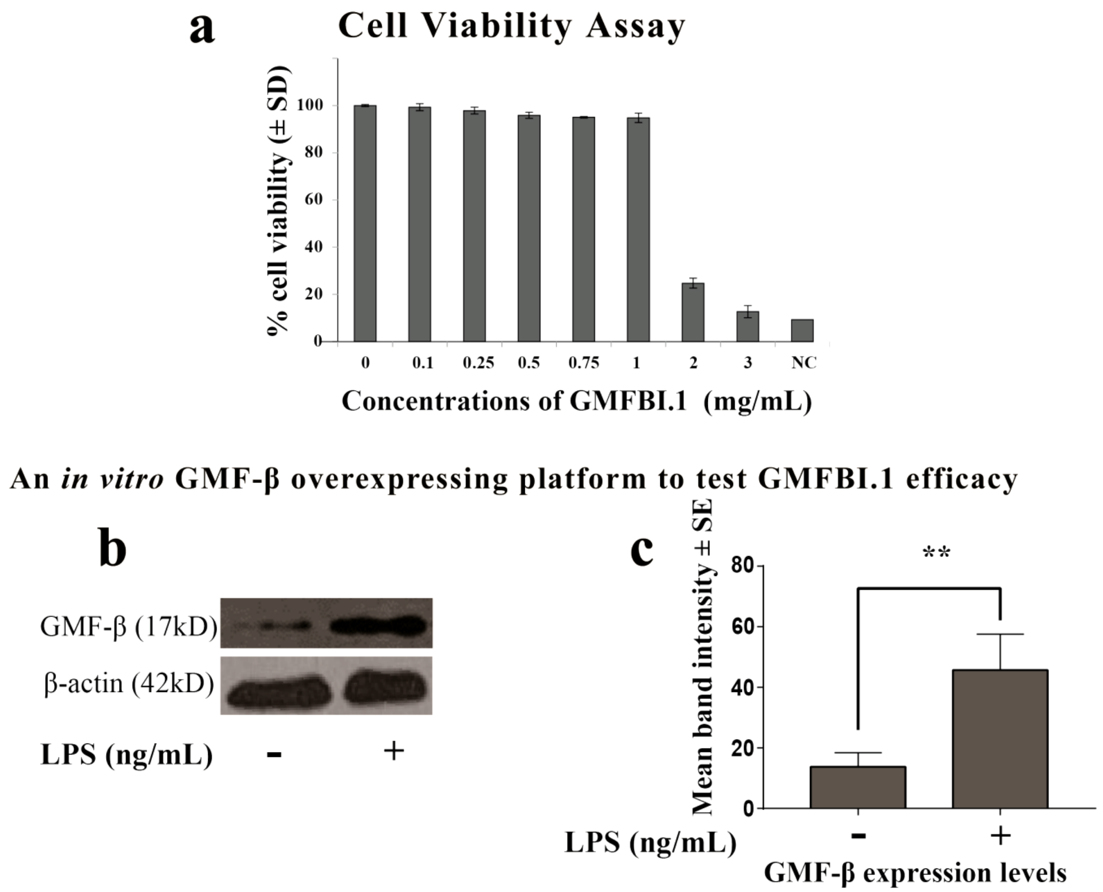

Figure S6. GMFBI.1 is non-toxic to the cells at its ED_{50} values and development of GMF-β overexpressing platform for testing GMFBI.1 efficacy (a) Cell viability of astrocytes following incubation with increasing concentrations of GMFBI.1 for 48 hrs, NC: negative control (10% Triton X-100). **(b)**Generation of GMF-β overexpression model *in vitro* in astrocytes as seen in EAE following stimulation with LPS in presence (+; 100 ng/mL) and its absence (-). **(c)** quantitative comparison of GMF-β expression following LPS stimulation of astrocytes (n=3, ** $p<0.01$).

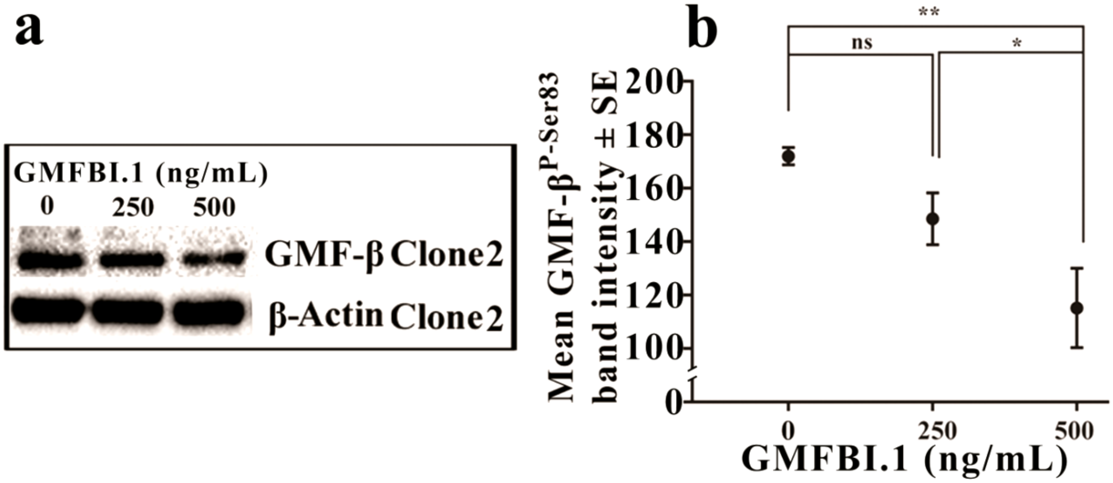

Figure S7. Effect of different concentrations of GMFBI.1 on Ser83 hGMF-β phosphorylation in different hGMF-β expressing stable clones of astrocytes. (a) Representative stably expressing GMF-β clone showing decrease in Ser83 phosphorylation following incubation with different concentrations (0, 250 and 500 ng/mL) of GMFBI.1 for 30min analysed by immunoblotting using anti-phospho Ser83 antibody. **(b)** Note the significant reduction in Ser83 phosphorylation on GMF-β following treatment with 500ng GMFBI.1 compared to 250ng (n=3, $p< 0.0183$ vs 250 ng; ** $p < 0.0013$ vs untreated). However, 250 ng incubation showed a low potency in Ser83 phosphorylation [*p value* not significant (ns) vs untreated] compared to 500ng indicating that with increasing concentration of the compound, increased blocking potency of the Ser83 phosphorylation could be seen.

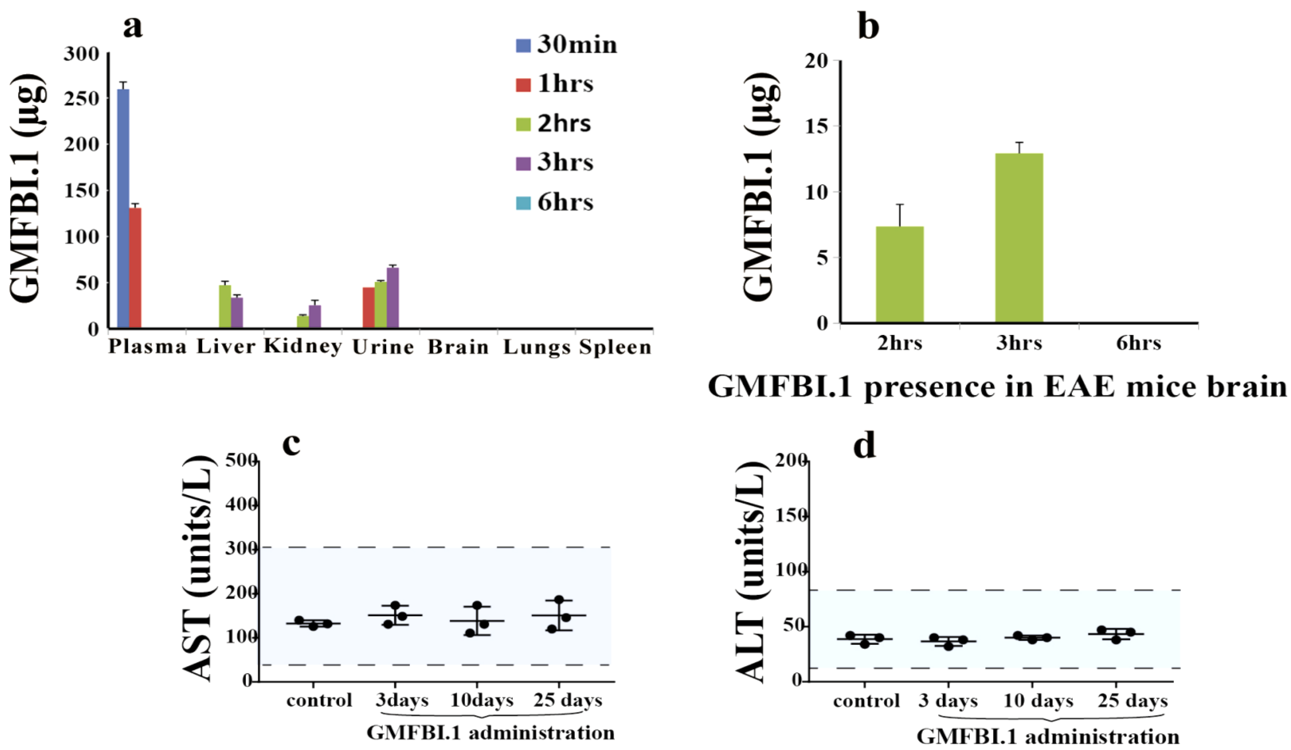

Figure S8. *In vivo* toxicity and bio-distribution of GMFBI.1. (a). Amounts ofGMFBI.1 in different organs, plasma and urine of normal healthy mice at different time intervals (30 minutes,1,2,3 and 6 hours) (n=3 per group). Note that by 6 hr, GMFBI.1 is undetectable in any organs especially in the brain. (**b).** Detection of GMFBI.1 in the brain of EAE animals and by 3 hr maximum concentration was observed. (**c&d)**. Note the non-toxicity of GMFBI.1 with respect to AST and ALT liver enzymes in GMFBI.1 administered (14 mg/kg twice daily) mice compared to saline controls at 3, 10 and 25 days (n=3 per group). Grey shaded region denotes normal activity range of AST and ALT enzymes in mice.

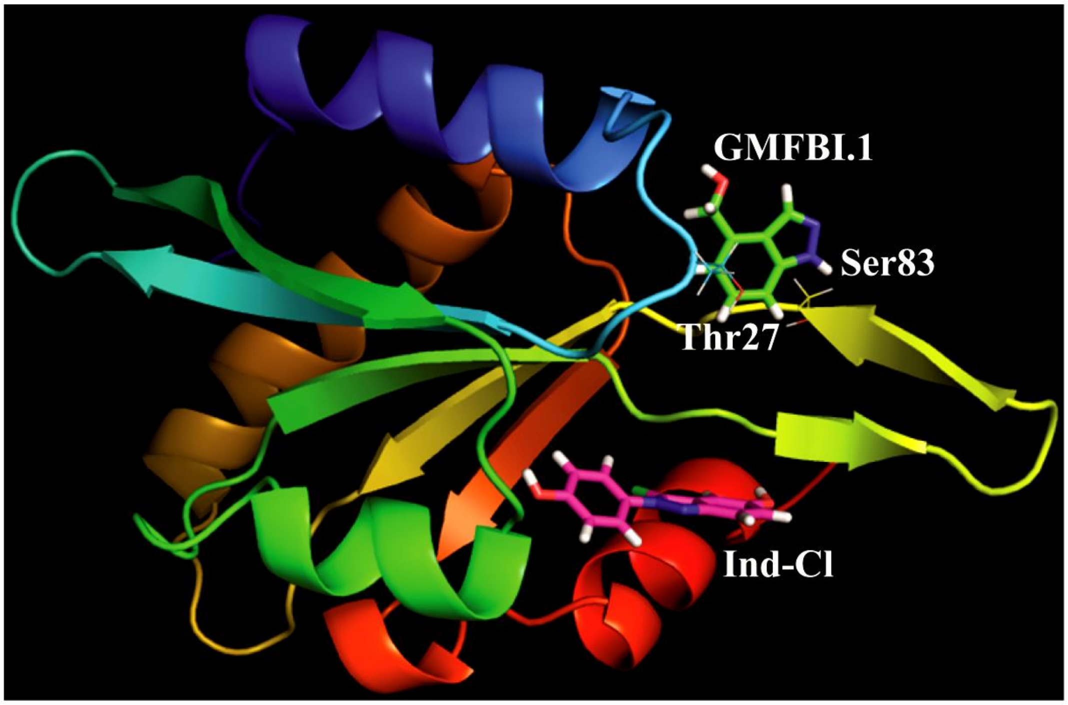

Figure S9. GMFBI.1 and Ind-Cl binding sites on hGMF- β. Binding site of Ind-Cl compound is outside the active site of hGMF-β, showing its non-specific binding with respect to that of GMFBI.1.

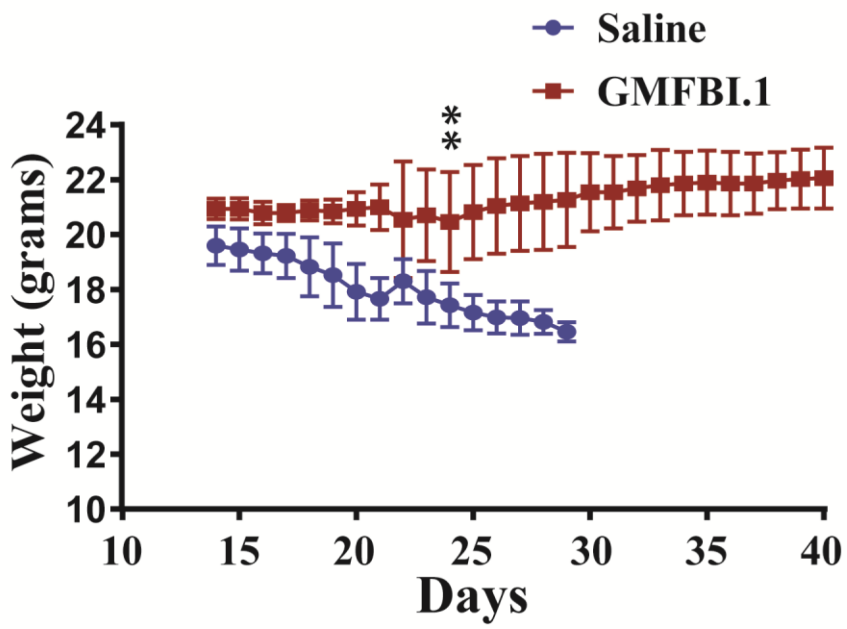

Figure S10. Weight Gain in EAE mice following GMFBI.1 treatment. Significant weight gain was observed in animals of GMFBI.1 treated group compared to the saline treated EAE control after 11 days of treatment (** $p<0.001$).

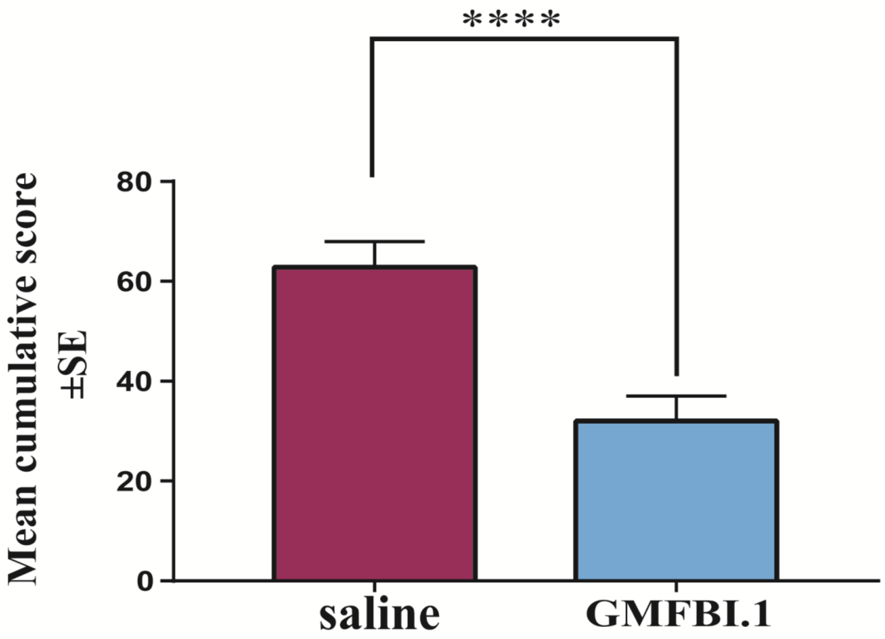

Figure S11. Mean cumulative clinical score of animals treated with GMFBI.1 compared with saline treated controls from day 15 to 29 post immunization (**** $p < 0.0001$).

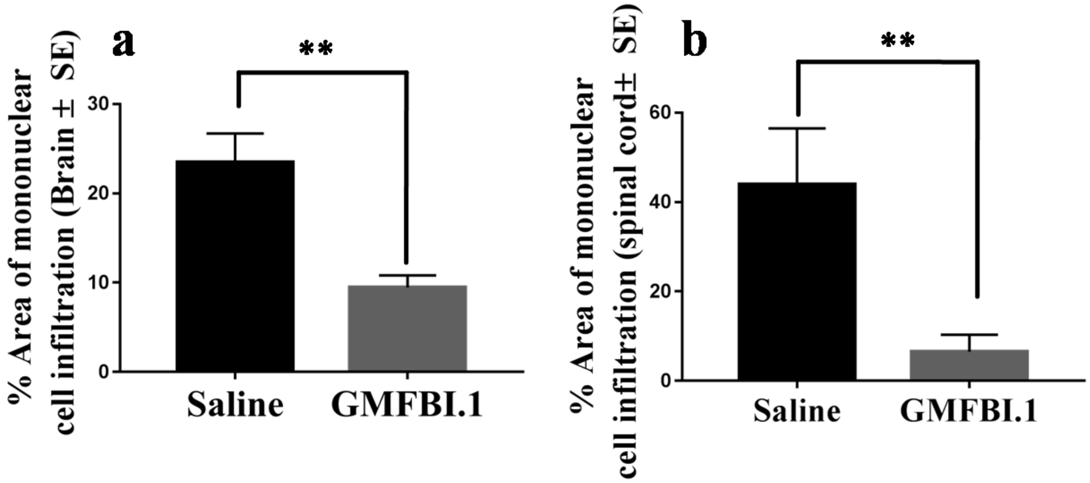

Figure S12. Quantification of infiltrated mononuclear cells in brain and spinal cord before and after GMFBI.1 treatment of EAE mice. Percentage area of mononuclear cell infiltration in both brain and spinal cord of saline and GMFB1.1 treated EAE mice were quantified from several randomly distributed 20X fields of (a) brain and (b)spinal cord areas of saline and GMFB1.1 treated mice (n=3 animals per group). Note the significant reduction in the mononuclear cells in GMFBI.1 treated EAE mice compared to saline treated controls (**p < 0.001).

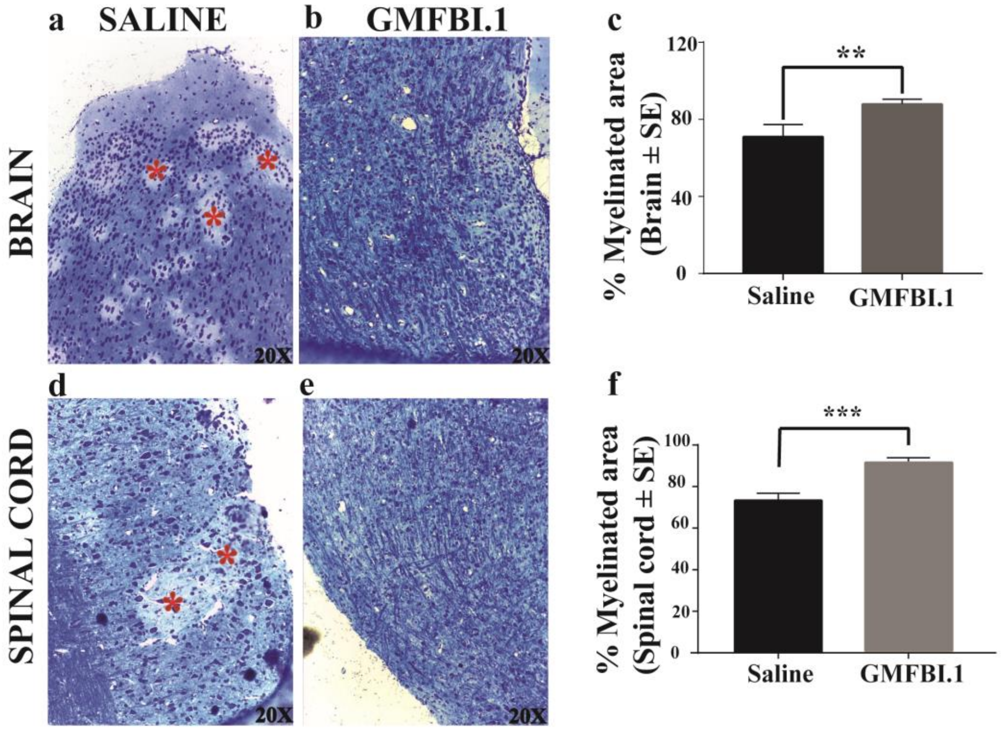

Figure S13. **GMFB1.1 treatment leads to increased myelination in the CNS of EAE mice**. Representative 20X microscopic fields of brain and spinal cord sections of saline and GMFB1.1 treated mice stained with Luxol fast blue stain is shown (a,b,d,e). Asterisk (*) indicates the demyelinated areas. Histogram quantifies the percentage myelination in GMFBI.1 treated mice vs saline treated EAE mice [**p< 0.001 vs saline in brain (c), ***p< 0.0001 vs saline in spinal cord (f)].

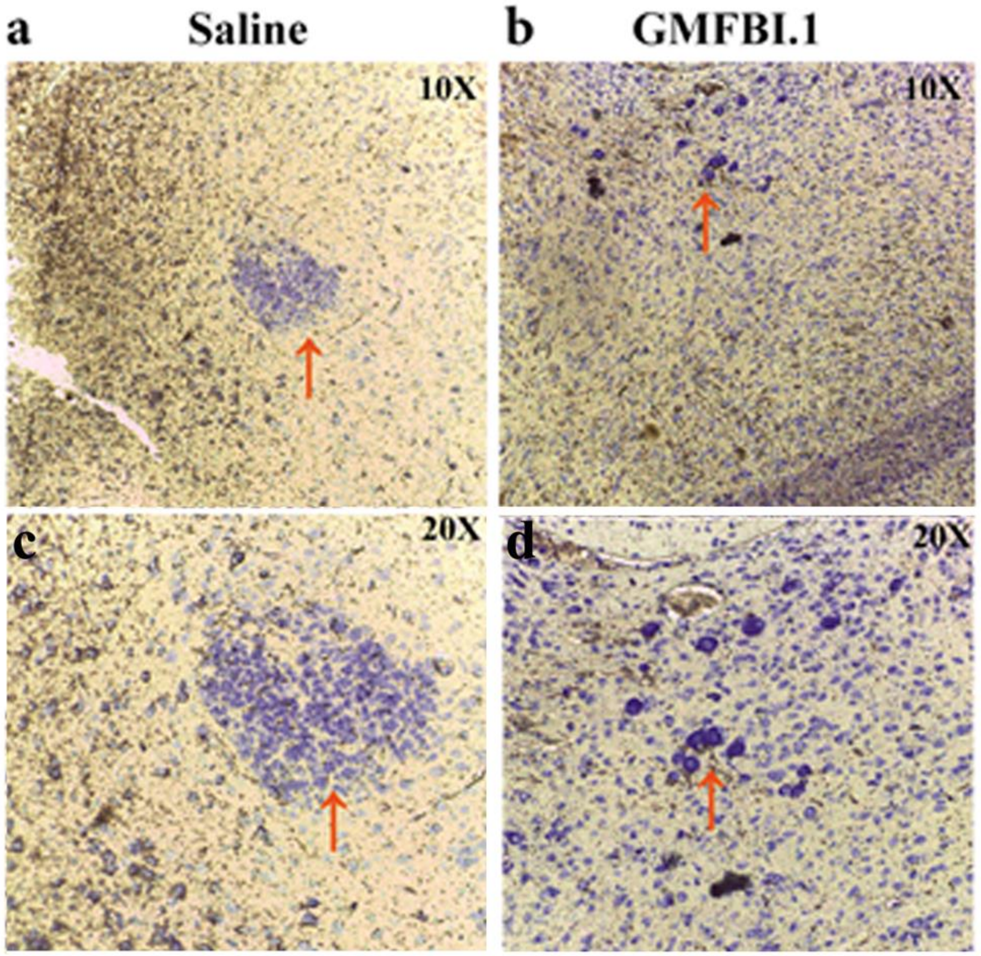

Figure S14. GMFB1.1 treatment leads to reduced mast cell infiltration in the CNS of EAE mice. Representative 10X and 20X microscopic images of brain sections of saline (a, c) and GMFB1.1(b, d) treated mice stained with toluidine blue is shown (arrows indicates stained mast cells). Reduced number of mast cells was seen in the sections of animals treated with GMFBI.1 compared to saline treated EAE controls.

a

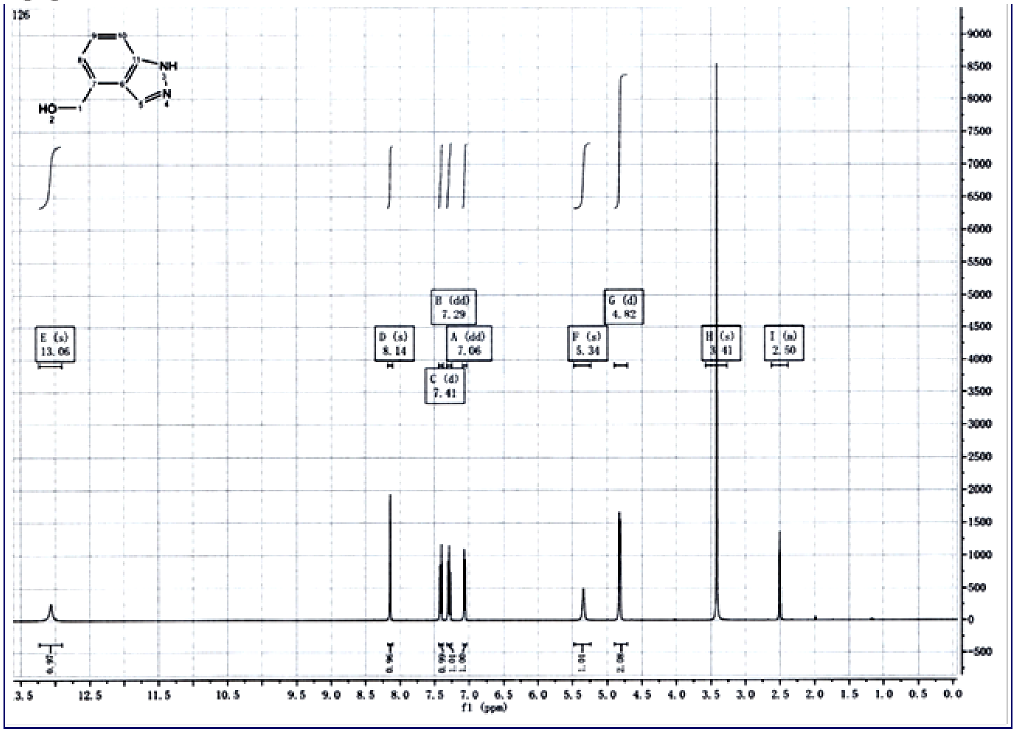

b

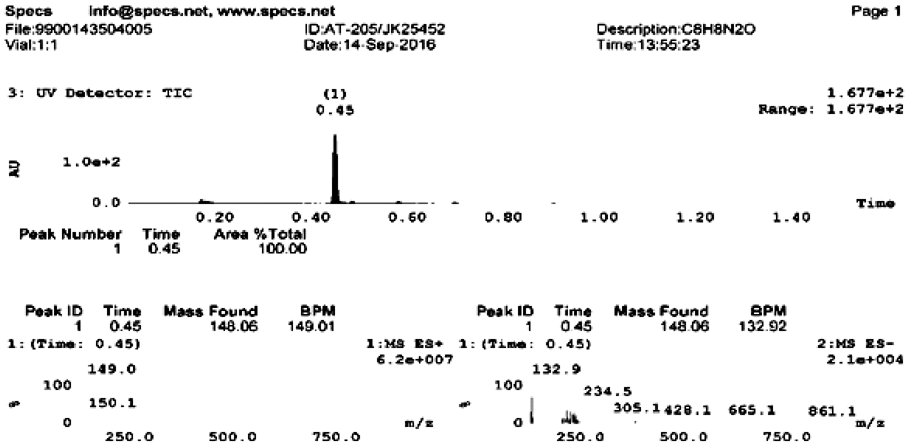

Figure S15. NMR spectrum analysis and HPLC Chromatogram of GMFBI.1. (a). Standard peak listings for ^{1}H NMR and proton-decoupled ^{13}C NMR of (1H-indazol-4-yl) methanol (GMFBI.1) **(b).** Chromatogram showing the HPLC analysis of (1H-indazol-4-yl) methanol (GMFBI.1) having > 95% purity. Note the appearance of a single peak at 0.45 retention time.

Movie S1. EAE animal before treatment with GMFBI.1. A representative video of an EAE animal severely paralyzed following induction of EAE.

Movie S2. EAE animal after treatment with GMFBI.1. The same EAE animal shown in movie S1following treatment with GMFBI.1 for 25 days. Note the significant improvement in ambulatory capacity of GMFBI.1 treated animal.

Table S1. ADME/T prediction of small molecule drugs for MS in comparison with GMFBI.1

Name (Status/Target)	Structure	CNS Activity	M.W (Da)	QPlog HERG	QPP Caco	QPlogBB	PHOA
**GMFBI.1** (Present study/ (EAE) GMF-β)	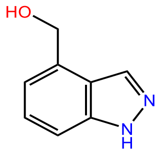	-1	148.2	-3.59	684.4	-0.51	81.4
**Ind-Cl** (EAE/ Estrogen receptor)	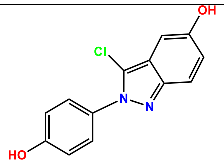	-1	260.7	-5.06	646.6	-0.53	92.3
**Dimethyl fumarate** (Phase III/ Unknown immune-modulator)	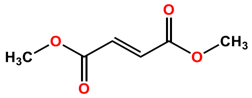	0	144.1	-3.52	434.4	-0.84	74.3
**Teriflunomide** (Phase III / MS target unknown)	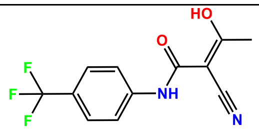	0	270.2	-4.98	1153.3	-0.36	92.6
**Fingolimod** (Prescribed drug/ Sphingosine-1 receptor)	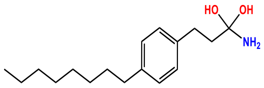	-2	313.5	-5.62	152.6	-1.298	83.9
**Clemastine** (Antihistamine/ Histamine antagonist)	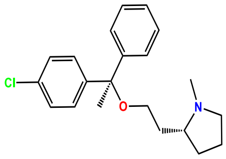	1	343.9	-7.01	2470.6	-0.475	100
**14-dehydro ergosterol**	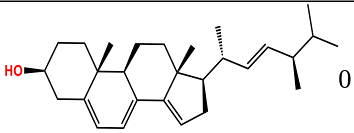	0	394.6	-4.73	3428.6	-0.209	100
**Rivaroxaban**	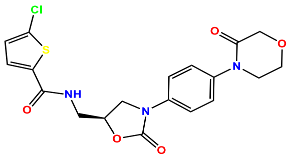	-1	435.8	-5.16	428.4	-0.754	85.1

MW:Molecular weight; **CNS=** predicted CNS activation**; QPlogBB=** Predicted brain/blood barrier partition coefficient; **Percent Human-Oral Absorption (PHOA); QPlogHERG=** Predicted IC_{50} value for blockage of HERG K^+ channels (Cardiotoxicity). **QPPCaco=** Predicted apparent Caco-2 cell permeability in nm/sec. Caco-2 cells are a model for the gutblood barrier. QikProp predictions are for

non-active transport. None of the VS hits go outside the recommended ranges of the above mentioned six ADMET parameters.
